# Genome Wide Association Identifies Common Variants at the *SERPINA6/SERPINA1* Locus Influencing Plasma Cortisol and Corticosteroid Binding Globulin

**DOI:** 10.1371/journal.pgen.1004474

**Published:** 2014-07-10

**Authors:** Jennifer L. Bolton, Caroline Hayward, Nese Direk, John G. Lewis, Geoffrey L. Hammond, Lesley A. Hill, Anna Anderson, Jennifer Huffman, James F. Wilson, Harry Campbell, Igor Rudan, Alan Wright, Nicholas Hastie, Sarah H. Wild, Fleur P. Velders, Albert Hofman, Andre G. Uitterlinden, Jari Lahti, Katri Räikkönen, Eero Kajantie, Elisabeth Widen, Aarno Palotie, Johan G. Eriksson, Marika Kaakinen, Marjo-Riitta Järvelin, Nicholas J. Timpson, George Davey Smith, Susan M. Ring, David M. Evans, Beate St Pourcain, Toshiko Tanaka, Yuri Milaneschi, Stefania Bandinelli, Luigi Ferrucci, Pim van der Harst, Judith G. M. Rosmalen, Stephen J. L. Bakker, Niek Verweij, Robin P. F. Dullaart, Anubha Mahajan, Cecilia M. Lindgren, Andrew Morris, Lars Lind, Erik Ingelsson, Laura N. Anderson, Craig E. Pennell, Stephen J. Lye, Stephen G. Matthews, Joel Eriksson, Dan Mellstrom, Claes Ohlsson, Jackie F. Price, Mark W. J. Strachan, Rebecca M. Reynolds, Henning Tiemeier, Brian R. Walker, on behalf of the CORtisol NETwork (CORNET) Consortium

**Affiliations:** 1 University/BHF Centre for Cardiovascular Science, Queen's Medical Research Institute, University of Edinburgh, Edinburgh, United Kingdom; 2 MRC Human Genetics Unit, Institute for Genetics and Molecular Medicine, University of Edinburgh, Edinburgh, United Kingdom; 3 Department of Epidemiology, Erasmus Medical Centre, Rotterdam, The Netherlands; 4 Canterbury Health Laboratories, Christchurch, New Zealand; 5 Department of Cellular and Physiological Sciences, Life Sciences Institute, University of British Columbia, Vancouver, Canada; 6 Centre for Population Health Sciences, Institute for Genetics and Molecular Medicine, University of Edinburgh, Edinburgh, United Kingdom; 7 Institute of Behavioural Sciences, University of Helsinki, Helsinki, Finland; 8 National Institute for Health and Welfare, Helsinki, Finland; 9 Institute for Molecular Medicine Finland (FIMM), University of Helsinki, Helsinki, Finland; 10 Department of Medical Genetics, University of Helsinki and University Central Hospital, Helsinki, Finland; 11 Department of General Practice and Primary Health Care, University of Helsinki, Helsinki, Finland; 12 Helsinki University Central Hospital, Unit of General Practice, Helsinki, Finland; 13 Folkhalsan Research Centre, Helsinki, Finland; 14 Vasa Central Hospital, Vasa, Finland; 15 Institute of Health Sciences and Biocenter Oulu, University of Oulu, Oulu, Finland; 16 Department of Children and Young People and Families, National Institute for Health and Welfare, Oulu, Finland; 17 Department of Epidemiology and Biostatistics, MRC-HPA Centre for Environment and Health, Imperial College London, London, United Kingdom; 18 Unit of Primary Care, Oulu University Hospital, Oulu, Finland; 19 MRC Centre for Causal Analyses in Translational Epidemiology, School of Social and Community Medicine, University of Bristol, Bristol, United Kingdom; 20 School of Social and Community Medicine, University of Bristol, Bristol, United Kingdom; 21 Longitudinal Studies Section, Clinical Research Branch, National Institute on Aging, Baltimore, Maryland, United States of America; 22 Department of Psychiatry, VU University Medical Center/GGZ inGeest, Amsterdam, The Netherlands; 23 Geriatric Unit, ASF, Florence, Italy; 24 University of Groningen, University Medical Center Groningen, Department of Cardiology, Groningen, The Netherlands; 25 University of Groningen, University Medical Center Groningen, Department of Genetics, Groningen, The Netherlands; 26 Durrer Center for Cardiogenetic Research, ICIN-Netherlands Heart Institute, Utrecht, The Netherlands; 27 University of Groningen, University Medical Center Groningen, Interdisciplinary Center for Psychiatric Epidemiology, Groningen, The Netherlands; 28 University of Groningen, University Medical Center Groningen, Department of Internal Medicine, Groningen, The Netherlands; 29 Wellcome Trust Centre for Human Genetics, University of Oxford, Oxford, United Kingdom; 30 Department of Medical Sciences, Uppsala University, Uppsala, Sweden; 31 Samuel Lunenfeld Research Institute, Mount Sinai Hospital, Toronto, Ontario, Canada; 32 School of Women's and Infant's Health, The University of Western Australia, Crawley, Australia; 33 Department of Physiology, University of Toronto, Toronto, Ontario, Canada; 34 Center for Bone and Arthritis Research, Institute of Medicin, Sahlgrenska Academy, University of Gothenburg, Gothenburg, Sweden; University of Michigan, United States of America

## Abstract

Variation in plasma levels of cortisol, an essential hormone in the stress response, is associated in population-based studies with cardio-metabolic, inflammatory and neuro-cognitive traits and diseases. Heritability of plasma cortisol is estimated at 30–60% but no common genetic contribution has been identified. The CORtisol NETwork (CORNET) consortium undertook genome wide association meta-analysis for plasma cortisol in 12,597 Caucasian participants, replicated in 2,795 participants. The results indicate that <1% of variance in plasma cortisol is accounted for by genetic variation in a single region of chromosome 14. This locus spans *SERPINA6*, encoding corticosteroid binding globulin (CBG, the major cortisol-binding protein in plasma), and *SERPINA1*, encoding α1-antitrypsin (which inhibits cleavage of the reactive centre loop that releases cortisol from CBG). Three partially independent signals were identified within the region, represented by common SNPs; detailed biochemical investigation in a nested sub-cohort showed all these SNPs were associated with variation in total cortisol binding activity in plasma, but some variants influenced total CBG concentrations while the top hit (rs12589136) influenced the immunoreactivity of the reactive centre loop of CBG. Exome chip and 1000 Genomes imputation analysis of this locus in the CROATIA-Korcula cohort identified missense mutations in *SERPINA6* and *SERPINA1* that did not account for the effects of common variants. These findings reveal a novel common genetic source of variation in binding of cortisol by CBG, and reinforce the key role of CBG in determining plasma cortisol levels. In turn this genetic variation may contribute to cortisol-associated degenerative diseases.

## Author Summary

Cortisol is a steroid hormone from the adrenal glands that is essential in the response to stress. Most cortisol in blood is bound to corticosteroid binding globulin (CBG). Diseases causing cortisol deficiency (Addison's disease) or excess (Cushing's syndrome) are life-threatening. Variations in plasma cortisol have been associated with cardiovascular and psychiatric diseases and their risk factors. To dissect the genetic contribution to variation in plasma cortisol, we formed the CORtisol NETwork (CORNET) consortium and recruited collaborators with suitable samples from more than 15,000 people. The results reveal that the major genetic influence on plasma cortisol is mediated by variations in the binding capacity of CBG. This is determined by differences in the circulating concentrations of CBG and also in the immunoreactivity of its ‘reactive centre loop’, potentially influencing not only binding affinity for cortisol but also the stability of CBG and hence the tissue delivery of cortisol. These findings provide the first evidence for a common genetic effect on levels of this clinically important hormone, suggest that differences in CBG between individuals are biologically important, and pave the way for further research to dissect causality in the associations of plasma cortisol with common diseases.

## Introduction

The adrenal steroid hormone cortisol plays a vital role in adaptation to environmental stress. In response to stressors such as starvation, infection or injury, cortisol secretion is elevated by activation of the hypothalamic-pituitary-adrenal (HPA) axis. Cortisol acts predominantly through glucocorticoid receptors to induce a wide range of physiological responses, including liberating fuel (by facilitating gluconeogenesis and lipolysis), maintaining cardiovascular homeostasis (by inducing sodium retention and vasoconstriction), altering mood and memory (in favour of focusing on ‘fight or flight’ responses), and acting as a ‘brake’ on the innate immune response (preventing bystander damage from unrestrained inflammation) [1]. Chronic elevations in cortisol, however, may be maladaptive, as exemplified in patients with tumours of the pituitary or adrenal gland causing Cushing's syndrome; here, elevated plasma cortisol is responsible for obesity, type 2 diabetes, hypertension, dyslipidaemia, depression, memory loss, impaired wound healing, osteoporosis, myopathy, and many other features.

Epidemiological data suggest that subtle activation of the HPA axis associates with many of these traits within the population, in people who do not harbour the tumours which cause overt Cushing's syndrome. In these studies higher plasma cortisol concentration, measured in the morning, provided a robust marker of the activation of the HPA axis which accompanies high blood pressure, hyperglycaemia and dyslipidaemia [2]–[5], age-associated cognitive dysfunction [6], and low mood [7]. Conversely, lower cortisol associates with immunological abnormalities [8], post-traumatic stress disorder (PTSD) [9], and obesity [1] (the inverse association with obesity is likely due to increased metabolic clearance of cortisol and confounds the positive association of cortisol with other cardiovascular risk factors, explaining some inconsistencies in the associations of cortisol with ‘metabolic syndrome’ [1]). Mechanisms underlying these associations remain uncertain, with most investigators suggesting abnormal central control of the HPA axis [1], [10], [11]. A high proportion of cortisol in plasma is protein bound, mostly to corticosteroid binding globulin (CBG). Although variations in total CBG concentrations have been associated with features of metabolic syndrome [12], [13], this does not account entirely for associations of total plasma cortisol with other quantitative traits [5], [14], [15].

Morning plasma cortisol has a heritability of 30–60% [16]–[18]. Identifying genetic variants which contribute to variation in morning cortisol values could provide key insights into the mechanism of HPA axis activation associated with common quantitative traits, and an opportunity to dissect causality using Mendelian randomisation [19]. Attempts to identify these genetic variants to date have been limited to small candidate gene studies [18]. We therefore established the CORtisol NETwork (CORNET) consortium with the initial aim of identifying genetic determinants of inter-individual variation in HPA axis function.

## Results

### Genome-wide association meta-analysis

**Figure 1:**
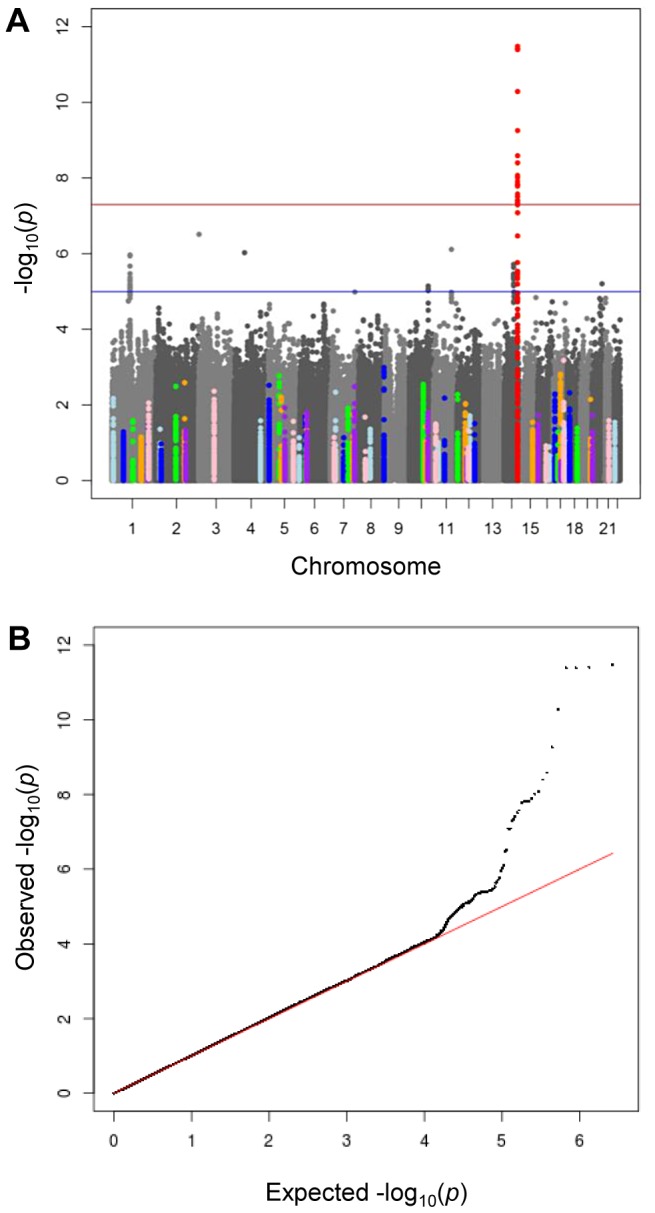
Meta-analysis of genome wide association studies for morning plasma cortisol. A) Manhattan plot of −lop_10_ *P* values by chromosome. The red horizontal line indicates genome-wide significance (*P*<5×10^−8^) and the blue horizontal line indicates moderate significance (*P*<5×10^−5^). The lead SNP rs12589136 (chr14:94,793,686; b37) in red is genome-wide significant. SNPs within ±50 kb of cortisol-related candidate genes (listed in Table S6) are highlighted in colours. B) Quantile-quantile plot of −log_10_ *P*, comparing the distribution of observed −log_10_ *P*-values and that expected by chance.

We conducted a discovery meta-analysis of genome-wide association studies (GWAMA) of morning plasma cortisol levels, investigating ∼2.5 M SNPs in 12,597 men and women, aged 14–102 years, of European origin (Table S1 for participant characteristics). There was very little inflation of test statistics (λ_GC_ = 1.005, Table S2). The −log_10_ *P* values by chromosome for age- and sex-adjusted cortisol z-scores are shown in Figure 1a. A quantile–quantile plot (Figure 1b) showed marked departure from the null for SNPs with low *P* values, listed in Table S3. Analysis of data for men and women separately showed no sex-specific effects (data not shown). The results were similar between all multivariable adjusted models, and whether or not time of sampling was included as a covariate. The results reported are therefore adjusted only for age and sex.

**Figure 2:**
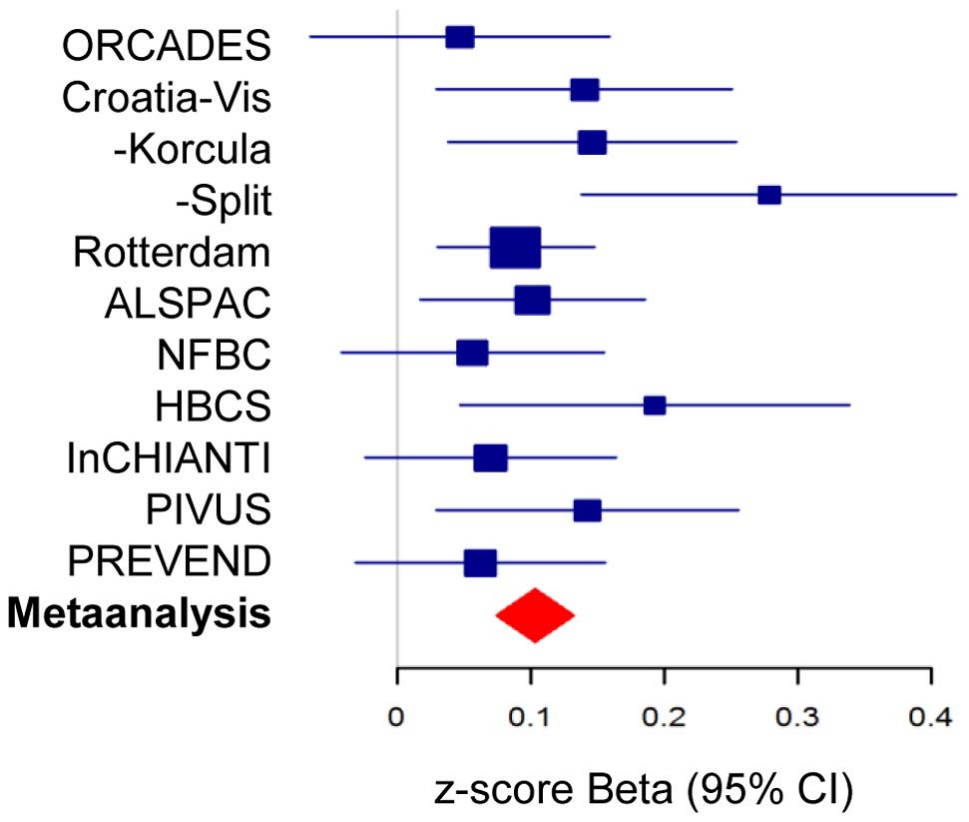
Forest plot of association of morning plasma cortisol with rs12589136. Plot shows association as beta values with 95%CI for morning plasma cortisol z-scores for rs12589136 (T allele) in discovery cohorts (blue) and meta-analysis (red).

There was strong evidence for associations between plasma cortisol and genetic variation found at chromosome 14q32. In an additive genetic model, the lead SNP rs12589136 reported a per minor allele effect of 0.10 cortisol z-score (95%CI 0.07,0.13; *P* = 4.0×10^−12^ after genomic control). The effect allele frequency was 0.22 and this variation explained 0.13% of the morning plasma cortisol variance. A forest plot showed consistent directional effects in all studies, with the T allele at rs12589136 associated with higher morning plasma cortisol (Figure 2). Only minimal heterogeneity was observed between studies (I^2^ = 0.18).

**Figure 3:**
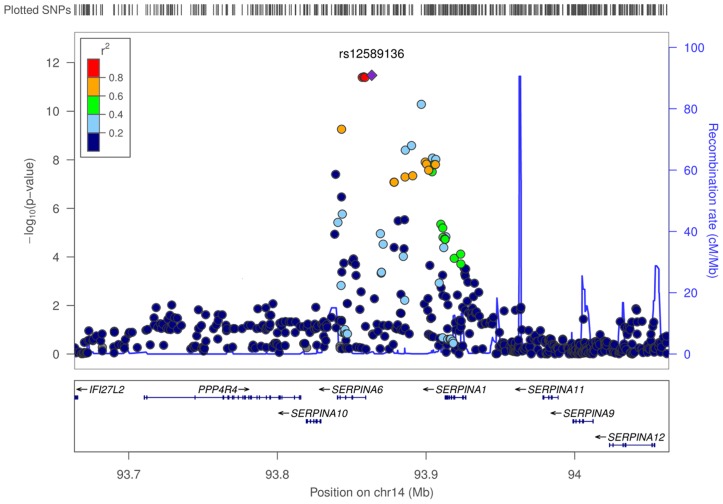
Regional associations surrounding lead SNP rs12589136 in genome-wide meta-analysis of morning plasma cortisol. Regional plot shows −log_10_ *P* values of all SNPs, and degree of correlation between all SNPs and lead SNP rs12589136. SNPs with lower *P* values span *SERPINA6* and *SERPINA1* genes within a recombination boundary.

A recombination boundary containing *SERPINA6* and *SERPINA1* was found to contain all variants at this locus contributing to association with plasma cortisol (Figure 3). A clumping procedure [20] identified rs12589136 (4 kb upstream of *SERPINA6*), rs11621961 (1 kb downstream of *SERPINA6*) and rs2749527 (30 kb upstream of *SERPINA6*) as markers representing genome-wide significant signals in this region. Individually, the beta (for effect on cortisol z score, 95%CI) for minor (all T) alleles at rs12589136, rs2749527, and rs11621961 were 0.10 (0.07,0.13; *P* = 3.3×10^−12^), −0.08 (−0.11,−0.06; *P* = 5.2×10^−11^), and −0.08 (−0.10,−0.05; *P* = 4.0×10^−8^); joint analysis showed these SNPs have partially independent effects, with beta (95%CI) 0.07 (0.04,0.10; *P* = 3.1×10^−5^), −0.04 (−0.07,−0.01; *P* = 0.012), and −0.03 (−0.07,0.00; *P* = 0.037), respectively.

The *SERPINA6* gene encodes corticosteroid binding globulin (CBG). The neighbouring (upstream) gene, *SERPINA1*, encodes α1-antitrypsin, the inhibitor of neutrophil elastase which cleaves and inactivates CBG [21].

### Conditional analysis

A quantile-quantile plot after removal of the *SERPINA6/SERPINA1* region (chr14: 94,768,859–94,843,565; Genome Reference Consortium build 37) showed no evident inflation of test statistics (not shown). Conditional analyses adjusting for each of the partially independent genome-wide significant variants (rs12589136, rs11621961, rs2749527) in a subset of the meta-analysis population reduced the significance of all other SNPs to *P*>1×10^−5^ for an association with plasma cortisol.

### Gene-centric associations

We used a gene-centric approach to analyse the combined effect of all SNPs within a gene, rather than individual SNP associations, using VEGAS [22]. This produced a gene-based test statistic from meta-analysis results, allowing identification of genes containing multiple SNPs that individually did not reach genome-wide significance (Table S4). Only *SERPINA6* and *SERPINA1* were identified as gene-wide significant (*P*<3×10^−6^); both included rs12589136 in the gene boundary.

### Candidate gene analysis

A list of 61 candidate genes thought likely to influence plasma cortisol was collated by a panel of experts. A Manhattan plot of the −log_10_ *P* values highlighted for SNPs in these candidate genes showed that only *SERPINA6* reached genome-wide significance (Figure 1a). Using gene-based p-values from VEGAS, after adjusting for multiple testing, only *SERPINA6* was associated with plasma cortisol (Table S5). *SERPINA1* was not included in the candidate gene list.

### Replication

In 2,795 participants in additional cohort studies, the association with plasma cortisol was replicated for the lead SNP rs12589136 (*P* = 0.0002), rs11621961 (*P* = 0.003) and rs2749529 (used as proxy for rs2749527, *P* = 0.019)(Table 1; Table S1 for participant characteristics; Table S6 for results in each cohort).

**Table 1:** Association with morning plasma cortisol of SNPs representing signals in the *SERPINA6/SERPINA1* region from meta-analyses of discovery genome-wide association studies and of replication studies.

					GWAMA (n = 12,597)				REPLICATION (n = 2,795)			
SNP ID	Chr	Number of supporting SNPs	Position (b37)	Alleles effect/other	EAF	Effects	Beta (95%CI)	*P^a^*	EAF	Effects	Beta (95%CI)	*P^a^*
rs12589136	14	30	94,793,686	T/G	0.22	+++++++++++	0.10 (0.07,0.13)	3.3×10^−12^	0.21	+++	0.12 (0.06,0.18)	0.0002^4^
rs2749527	14	17	94,827,068	T/C	0.49	-----------	−0.08 (−0.11,−0.06)	5.2×10^−11^			n/a	
rs2749529^b^	14	n/a	94,820,459	T/A	0.47	+++++++++++	0.07 (0.05,0.10)	2.6×10^−9^	0.45	+++	0.06 (0.01,0.11)	0.019
rs11621961	14	0	94,769,476	T/C	0.36	--------?—	−0.08 (−0.10,−0.05)	4.0×10^−8^	0.37	---	−0.08 (−0.14,−0.03)	0.003

a adjusted for age and sex.

b rs2749527 was replaced with rs2749529 (r^2^ = 0.905, D' = 1.0) as rs2749527 failed manufacture for replication. LD patterns (from SNAP HapMap CEU build 22): rs11621961-rs12589136 (r^2^ = 0.131), rs11621961-rs2749529 (r^2^ = 0.260), rs12589136-rs2749529 (r^2^ = 0.291), rs11621961-rs2749527 (r^2^ = 0.255). Independent SNPs were defined by PLINK using the clumping function (within 500kb, LD r^2^ >0.2, P-value <5x10^−5^)

### Functional consequences of genetic variation in the SERPINA6/SERPINA1 region

We explored the associations between rs12589136, rs11621961, rs2749527 with cortisol and CBG phenotypes in more detail in 316 subjects from the CROATIA-Korcula cohort (Table 2). Together these three SNPs explained 0.54% of the variance in total plasma cortisol in CROATIA-Korcula. However, there were distinct patterns of association of ‘high cortisol’ alleles with CBG. After adjusting for age and sex, although all three variants were associated with differences in total cortisol binding activity, measured by the binding of [^3^H]-cortisol, there were different associations with CBG immunoreactivity. The T allele at rs2749527 was associated with higher ‘total’ CBG concentration by radioimmunoassay, and there were similar, but weaker associations of total CBG immunoreactivity with variation at rs11621961. Differences in calculated free plasma cortisol reflected these differences in total CBG immunoreactivity, which is used in the calculation of free cortisol. In contrast, however, the minor (T) allele at rs12589136 was not associated with ‘total’ CBG immunoreactivity but was strongly associated with the proportion of CBG bound by a monoclonal antibody against an epitope in the reactive centre loop of CBG [23]. None of these SNPs representing signals in the *SERPINA6/SERPINA1* region was associated with α1-antitrypsin concentrations in blood. However, α1-antitrypsin levels were negatively correlated with plasma total cortisol (beta −0.17 (95%CI −0.28, −0.06); *P* = 0.002) and calculated free cortisol (beta −0.13 (95%CI −0.24, −0.02); *P* = 0.021), although they did not correlate with ratio of intact/total CBG 0.02 (95%CI −0.09, 0.13; *P* = 0.715).

**Table 2:** Functional consequences of variants in the *SERPINA6/A1* locus significantly associated with morning plasma cortisol in GWAMA, and of the Leuven variant, in CROATIA-Korcula.

	rs11621961			Beta (95%CI)	*P* c
	TT	CT	CC		
Total cortisol^a^	655 (618,694) [129]	664 (644,685)[411]	675 (654,696)[357]	−0.05 (−0.14,0.05)	0.305
Calculated free cortisol	42 (35.0,51)[56]	47 (42,52)[126]	50 (46,55)[136]	−0.14 (−0.28,0.00)	0.049
Measured free cortisol^b^	14 (11,17)[27]	14 (11,16)[70]	13 (10,16)[67]	0.01 (−0.2,0.22)	0.933
Total CBG	0.90 (0.82,0.99)[56]	0.90 (0.85,0.94)[126]	0.96 (0.92,1.01)[136]	−0.12 (−0.26,0.02)	0.103
RCL/total CBG	0.76 (0.71,0.80)[56]	0.77 (0.74,0.79)[125]	0.76 (0.73,0.78)[136]	0.01 (−0.14,0.15)	0.940
Cortisol binding activity	503 (473,532)[56]	492 (474,511)[125]	531 (512,550)[136]	−0.17 (−0.32,−0.03)	0.016
α1-antitrypsin	2.74 (2.37,3.16)[56]	2.74 (2.51,2.99)[124]	2.66 (2.43,2.91)[134]	0.02 (−0.14,0.17)	0.840

	rs12589136				
	TT	GT	GG		
Total cortisol^a^	706 (656,760)[53]	681 (657,706)[302]	655 (638,673)[540]	0.13 (0.02,0.24)	0.017
Calculated free cortisol	47 (41,54)[52]	54 (45,65)[47]	46 (42,50)[217]	0.13 (−0.01,0.26)	0.073
Measured free cortisol^b^	13 (11,16)[39]	n/a	13 (12,15)[125]	0.03 (−0.15,0.2)	0.777
Total CBG	1.01 (0.95,1.07)[52]	0.91 (0.82,1.02)[47]	0.91 (0.87,0.95)[217]	0.06 (−0.07,0.20)	0.371
RCL/total CBG	0.68 (0.64,0.71)[52]	0.69 (0.65,0.73)[47]	0.80 (0.78,0.82)[216]	−0.49 (−0.64,−0.34)	9.4×10^−11^
Cortisol binding activity	557 (532,581)[52]	552 (513,591)[47]	491 (477,505)[216]	0.30 (0.16,0.44)	2.3×10^−5^
α1-antitrypsin	2.68 (2.36,3.05)[51]	2.77 (2.43,3.16)[47]	2.69 (2.50,2.90)[214]	0.06 (−0.09,0.22)	0.421

	rs2749527				
	CC	CT	TT		
Total cortisol^a^	659 (635,684)[251]	668 (649,687)[431]	674 (643,707)[212]	0.03 (−0.06,0.12)	0.495
Calculated free cortisol	49 (43,55)[102]	45 (41,50)[123]	48 (42,55)[92]	0.02 (−0.11,0.16)	0.717
Measured free cortisol^b^	16 (14,19)[60]	11 (8,15)[51]	13 (11,15)[53]	−0.11 (−0.29,0.07)	0.246
Total CBG	0.87 (0.82,0.92)[102]	0.92 (0.87,0.98)[123]	1.00 (0.96,1.05)[92]	0.17 (0.03,0.30)	0.014
RCL/total CBG	0.76 (0.73,0.79)[102]	0.76 (0.74,0.79)[122]	0.76 (0.73,0.79)[92]	−0.02 (−0.16,0.13)	0.824
Cortisol binding activity	486 (468,504)[102]	507 (486,528)[122]	545 (524,566)[92]	0.24 (0.11,0.38)	4.3×10^−4^
α1-antitrypsin	2.58 (2.31,2.88)[101]	2.86 (2.62,3.12)[121]	2.65 (2.38,2.94)[91]	0.08 (−0.06,0.23)	0.268

	rs113418909 (Leuven)				
	AA	AT			
Total cortisol	673 (658,688)[792]	562 (500,631)[28]		−0.63 (−1.01,−0.25)	0.001
Calculated free cortisol	48 (45,52)[280]	38 (27,52)[9]		−0.43 (−1.06,0.20)	0.182
Measured free cortisol	14 (12,15)[147]	13 (8,20)[5]		−0.04 (−0.92,0.83)	0.924
Total CBG	0.93 (0.90,0.96)[280]	0.94 (0.82,1.07)[9]		0.05 (−0.58,0.68)	0.870
RCL/total CBG	0.76 (0.74,0.78)[279]	0.82 (0.78,0.87)[9]		0.51 (−0.18,1.19)	0.146
Cortisol binding activity	512 (500,524)[279]	347 (305,389)[9]		−1.54 (−2.17,−0.91)	1.9×10^−6^
α1-antitrypsin	2.67 (2.51,2.84)[277]	2.40 (1.91,3.03)[9]		−0.12 (−0.83,0.58)	0.732

a Data for total cortisol is from the whole Croatia-Korcula sample, n = 898.

b Samples selected for measured free cortisol assay were age and sex matched homozygotes at rs12589136.

c adjusted for age, sex, and first three principal components, using kinship matrix derived from GWAS data.

Data are geometric mean (95% CI)[n]. Cortisol values are nmol/L, CBG µmol/L, cortisol binding activity nmol/L, and α1-antitrypsin g/L. RCL = reactive centre loop.

We investigated exome chip data for this locus in all CROATIA-Korcula participants to identify non-synonymous variants in the *SERPINA6/SERPINA1* region associated with plasma cortisol. From 34 variants on the exome chip in this region (chr14:94,770,585–94,857,029, build 37), 9 were polymorphic in this sample (Table S7) but only two were associated with plasma cortisol: rs113418909 in *SERPINA6* (Leu^115^His in CBG, previously reported as the Leuven mutation associated with low total cortisol [24]); and rs28931570 in *SERPINA1* (Arg^63^Cys in α1-antitrypsin, not recognised as a disease-causing variant [25]). We also analysed 735 additional SNPs in the same region imputed from 1000 Genomes data in all CROATIA-Korcula participants but did not find any additional SNPs associated with plasma cortisol, using a *P* threshold of 0.05/735 = 7×10^−5^. The two rare variants rs113418909 and rs28931570 were in perfect linkage disequilibrium amongst participants with detailed biochemical phenotyping performed, so results are reported for rs113418909 only (Table 2). Prevalence of the Leuven variant in CROATIA-Korcula was higher than expected (MAF = 0.017, compared with MAF = 0.0046 in dbSNP). After adjusting for age, sex, and accounting for kinship, participants who were heterozygote for the Leuven variant had lower total cortisol and markedly lower total cortisol binding activity, but normal CBG immunoreactivity (Table 2).

After removing subjects with the rare Leuven variant (ie. heterozygotes), rs12589136 remained associated with total cortisol (0.15, 95%CI 0.04,0.26; *P* = 0.009), calculated free cortisol (0.17, 95%CI 0.02,0.32; *P* = 0.031), and the proportion of CBG bound by the reactive centre loop antibody (−0.45, 95%CI −0.60,−0.29; *P* = 2.8×10^−8^).

## Discussion

These results clearly attribute inter-individual differences in morning plasma cortisol amongst Europeans to genetic variation within a region on chromosome 14 containing the *SERPINA6* and *SERPINA1* genes. The association of this region with plasma cortisol was consistent across multiple cohorts and was observed not only in genome-wide meta-analysis of individual SNPs, but also in gene-based hypothesis-free analysis, and in a candidate gene analysis. Investigation of the functional consequences of genetic variation in this region in a genetic isolate population in Croatia indicates that the effects of variation at *SERPINA6* and *SERPINA1* on plasma cortisol are likely to be mediated through alterations in total cortisol binding by corticosteroid binding globulin (CBG). In part, this is determined by differences in total CBG concentrations, and in part in association with a previously unrecognised variability in the immunoreactivity of the reactive centre loop of CBG. Since the process of CBG cleavage by neutrophil elastase and resultant reconfiguration of the reactive centre loop is considered important in the release of bioavailable cortisol within target tissues [21], [26], this finding provides a novel insight into a biological pathway controlling cortisol action.

The diverse actions of cortisol, and the striking clinical consequences of glucocorticoid excess or deficiency, have led many investigators to propose a central role for variations in cortisol levels in determining common quantitative traits. However, cortisol has not been measured as widely in epidemiological cohort studies as many other phenotypes. This may reflect the perceived difficulty of obtaining samples at a fixed time of day and in un-stressed conditions, to avoid confounding effects. The CORNET consortium had to decline samples from many cohorts in which time of sampling was inadequately controlled, and even then there was high variability in plasma cortisol. Thus, although the variants we identified in the *SERPINA6/SERPINA1* region of chromosome 14 accounted for <1% of the variance in plasma cortisol, this signal may be obscured by substantial unmeasured confounding and measurement error and may comprise a considerable component of the estimated 30–60% heritability of plasma cortisol [16]–[18]. We identified 3 SNPs with partially independent effects on plasma cortisol. There may be a small degree of linkage disequilibrium between these SNPs, but they also show different associations with CBG biochemistry, suggesting that they represent independent effects. None of these 3 SNPs appears directly to affect CBG function; although rs12589136 is close to a consensus estrogen response element, there was no gender difference in its association with plasma cortisol.

Previous investigations of the genetic determinants of plasma cortisol (reviewed in [18]) have been underpowered candidate gene studies, including some which included a tandem repeat in intron 1 of *SERPINA6* [27], [28]. Interestingly, many of the genetic variants previously associated with cortisol, eg for glucocorticoid [1] and mineralocorticoid [29], [30] receptors, showed no signal whatsoever in the adequately powered GWAMA and candidate gene analysis conducted here.

Rare mutations in *SERPINA6* have been described which cause absent CBG protein or, more often, reduced affinity of CBG for cortisol [31]–[35]. Affected individuals have low total plasma cortisol but normal free plasma cortisol. However, they also have abnormal pulsatility of plasma cortisol, and non-specific symptoms including fatigue which are unresponsive to cortisol supplementation; features which have been attributed to abnormal function of CBG in delivering cortisol to target tissues, including in brain regions involved in negative feedback regulation of the HPA axis [21]. Although one of these mutations, A51V, has been found to be surprisingly prevalent (MAF>3%) amongst Chinese subjects [34], it has not been found in non-Asian populations and we did not find Caucasians carrying this mutation when tested by exome chip analysis. In cohort studies, plasma CBG concentrations have been associated with features of the metabolic syndrome [12], [13], [15] and one previous candidate gene study with >900 participants showed that SNPs in *SERPINA6*, including some identified as being associated with plasma cortisol in this GWAMA, were predictive of somatic symptoms [36]. We found evidence that genetic variation in the *SERPINA6/SERPINA1* region influences total plasma cortisol not only through changes in total CBG concentrations, but also in association with alterations in the immunoreactivity of the reactive centre loop of the CBG protein.

Cleavage of the reactive centre loop (RCL) of CBG by neutrophil elastase and inhibition of elastase activity by α1-antitrypsin has been recognised for more than 20 years [26]. However, the recent development of monoclonal antibodies which recognise the intact RCL of CBG has allowed this process to be studied *in vivo* for the first time [23]. Using these tools in samples from Croatia-Korcula has provided the novel insight that immunoreactivity of the RCL of CBG is variable in the population, and further that this is explained in part by genetic variations in the *SERPINA6/SERPINA1* region. It remains to be determined whether this difference in immunoreactivity of the RCL represents altered susceptibility to CBG cleavage. We show that a common variant (rs12589136) associated with impaired RCL antibody binding was associated with higher total plasma cortisol and higher cortisol binding activity. These observations are inconsistent with the interpretation that impaired RCL antibody binding represents enhanced RCL cleavage [23], given that cleaved CBG has a lower affinity than intact CBG for cortisol binding [37]. Alternatively, the altered immunoreactivity of the RCL epitope may represent resistance to cleavage and hence enhanced cortisol binding. It is possible that the genetically determined difference in the RCL epitope of CBG is associated with impaired negative feedback of the HPA axis due to reduced tissue delivery of cortisol by CBG, analogous with findings in CBG knockout mice [38]. Although we could not confirm associated elevation in free plasma cortisol concentrations, these measurements are notoriously unreliable, for example being similarly unhelpful in dissecting the consequences of CBG deficiency described above.

We found further evidence for the importance of CBG using exome chips in the genetic isolate population of Korcula in Croatia, where we discovered an unusually high prevalence of heterozygotes for the Leuven mutation in *SERPINA6* [24]. These individuals have lower total plasma cortisol despite normal total CBG concentrations, and we confirmed substantial reductions in total cortisol binding activity, without any difference in RCL antibody binding. The presence of the Leuven variant, however, did not account for the association of the top hit SNPs identified by GWAMA with plasma cortisol or CBG RCL antibody binding.

It is possible that a combination of alterations in CBG substrate as well as in neutrophil elastase level and/or activity may determine cleavage of CBG and tissue delivery of cortisol, especially in local sites of inflammation [21]. Intriguingly, we found inverse associations between levels of α1-antitrypsin, the inhibitor of neutrophil elastase, and plasma cortisol concentrations, consistent with instability of CBG resulting in HPA axis activation as proposed above; however, we could not identify a genetic influence on this relationship, or confirm its association with CBG RCL immunoreactivity. Specifically, we did not identify independent signals for *SERPINA6* and *SERPINA1* in conditional analysis, and the rare variant Leuven mutation was in linkage disequilibrium with the only rare variant we identified in *SERPINA1*. Recent studies have identified variants in *SERPINA1* that are associated with coronary artery calcification [39] and serum lipid profile [40], the latter represented by rs1303 which is in linkage disequilibrium with the top hit rs12589136 identified by GWAMA (r^2^ = 0.35). These findings are consistent with variation in the *SERPINA6/SERPINA1* locus affecting downstream actions of cortisol, but it remains unclear if an interaction exists between the variants at these two genes. Mutations in *SERPINA1* cause the syndrome of α1-antitrypsin deficiency, but we are not aware of any investigations of CBG or cortisol in these patients, and their HPA axis may be disturbed anyway by un-restrained neutrophil-mediated tissue damage. Rare variants in *SERPINA1* (notably rs112635299) have been associated with α1-globulin plasma protein levels, of which α1-antitrypsin is a major constituent, using GWAS with 1000 Genomes imputation [41]. However, in the CROATIA-Korcula cohort neither rs1303 (Table S7) nor SNPs imputed from 1000 Genomes (including rs112635299) were associated with plasma cortisol. More detailed phenotyping amongst participants with contrasting genotypes at the *SERPINA6/SERPINA1* region will be required to clarify the basis for altered interaction between the two gene products.

These findings emphasise the biological importance of plasma protein binding for steroid hormones, and are analogous to recent findings that a common variant in sex hormone binding globulin contributes to variation in total testosterone levels [42]. Given the consequences of altered binding protein function for steroid volume of distribution and clearance, and documented effects on HPA axis function [21], this is an important finding of itself. However, potentially of greater importance is the novel observation that a key protein domain of CBG, the reactive centre loop, is subject to inter-individual differences which are influenced by genetic variation and may constitute a novel influence on tissue steroid action.

## Materials and Methods

### Gene discovery

We performed a meta-analysis of genome-wide association studies of morning plasma cortisol in 12,597 subjects from 11 western European population-based cohorts: CROATIA-Vis (n = 885), CROATIA-Korcula (n = 898), CROATIA-Split (n = 493), ORCADES (n = 886), Rotterdam Study (n = 2945), NFBC1966 (n = 1195), Helsinki Birth Cohort Study 1934–44 (n = 451), ALSPAC (n = 1567), InChianti (n = 1207), PREVEND (n = 1151), and PIVUS (n = 919). Replication was tested in 2,795 subjects from three independent cohorts: Raine Study (n = 797), ET2DS (n = 1,069), and MrOS-Sweden (n = 929). Cortisol was measured by immunoassay in blood samples collected from study participants between 0700 and 1100 h. Inclusion criteria were adults aged 17 years or older from Caucasian populations; exclusion criteria were current glucocorticoid use, pregnant or breast feeding women, and twins (exclusion of one). Characteristics of the study populations are presented in Table S1 and details of each cohort are provided in Text S1. All participants provided written informed consent and studies were approved by local Research Ethics Committees and/or Institutional Review Boards.

#### Association analysis with morning plasma cortisol

Each study performed single marker association tests, and study-specific linear regression models which used z-scores of log-transformed cortisol, additive SNP effects, and were adjusted for age and sex (model 1); age, sex, and smoking (model 2); or age, sex, smoking and body mass index (model 3). Imputation of the gene-chip results used the HapMap CEU population, build 36. In cohorts with consanguineous populations (ORCADES and Croatia), adjustments for principal components in kinship matrices were performed using ProbABEL; for other cohorts, Identity-By-Descent coefficients were calculated using PLINK and related participants excluded. In the majority of cohorts, participants were only included if blood samples had been obtained within a 60 minute time interval, when variations in time of sampling were ignored. In a subset of cohorts, samples were obtained over a wider time interval (but always in the morning before 1100 h) and time of blood sampling recorded; for these cohorts, three further models were run as above but also including time of sampling, calculated as minutes from first sampling time, as an additional covariate [43].

Quality control was carried out on the imputed genome-wide data for all 11 studies prior to meta-analysis; this excluded all samples with a minor allele frequency (MAF) <2%, call rate <95%, Hardy-Weinberg equilibrium (HWE)<1×10^−8^ and poor imputation quality (MACH R2_HAT<0.30, IMPUTE PROPER_INFO<0.60, BEAGLE INFO<0.30, as appropriate). Quantile-quantile (QQ) plots and genomic control (lambda) were used to confirm quality control. Sex chromosomes were not analysed.

#### Meta-analysis of association results

We performed fixed effects meta-analysis, which used combined allelic effects weighted by the inverse of their variance for each of the models using the GWAMA program [44]. This aligned all studies to the same reference allele at each SNP, thus avoiding strand errors, and excluded SNPs with obvious input errors (eg. discrepancies in effect allele frequencies). The results from analysis with or without genomic control were nearly identical, as expected with λ_GC_ = 1.005. The genome-wide significance threshold for the meta-analysis was *P*<5×10^−8^. Percentage variation of cortisol was calculated from meta-analysis results as (2*effect allele frequency)*(1-effect allele frequency)*(beta^2^/sd^2^). A regional plot was generated using LocusZoom [45], and heat map using snp.plotter [46] in R version 2.15.2. Joint analysis of meta-analysis was performed with the GCTA program [47].

#### Clumping analysis

To detect independent top SNPs on the basis of empirical estimates of linkage disequilibrium between the SNPs, we used the clumping function as implemented in PLINK [20]. All the SNPs with a *P*-value<5×10^−5^ in meta-analysis were used for clumping. We grouped the SNPs within 500 kb of the index SNP that have r^2^ >0.2 with the index SNP.

#### Replication genotyping and analysis

Genes identified in the meta-analysis were evaluated in the Raine Study, MrOS-Sweden, and Edinburgh type 2 Diabetes Study (ET2DS). Raine Study and MrOS-Sweden had GWAS data so we extracted the replication SNP results, and ET2DS was genotyped at the Wellcome Trust Clinical Research Facility Genetics Core Laboratory in Edinburgh using the OpenArray genotyping platform. rs2749527 failed manufacture for the OpenArray, and was replaced with rs2749529 (r^2^ = 0.905, D′ = 1.0) in all replication cohorts. Genotypic association analysis in these studies followed the same methods as those described above for the discovery meta-analysis, adjusting for age and sex.

#### Conditional & sex-specific analysis

We performed meta-analyses of sex-specific GWAS and conditional GWAS in a subset of populations, using single marker association tests of z-scores of log-transformed cortisol. For the sex-specific analysis, each study adjusted for age in both men (n = 3,546) and women (n = 5,956). For the conditional analysis, each study adjusted for age, sex, and each of the *SERPINA6* loci SNPs rs12589136 (n = 9,308), rs11621961 (n = 7,687), and rs2749527 (n = 9,307) individually. We then did fixed effects meta-analysis using GWAMA program.

#### Gene-based analysis

We used the Versatile Gene-based Association Study (VEGAS) program [22] to perform gene-centric analysis. This used individual SNP p-values derived from the meta-analysis results to compute a gene-based p-value. We used two methods: all SNPs within a gene, or a subset of the 10% most significant SNPs in each gene boundary. VEGAS accounted for linkage disequilibrium between SNPs using the HapMap phase 2 population (CEU). SNPs were assigned to ∼18,000 genes based on positions in build 36 (hg18), with gene boundaries of ±50 kb of the UTR. Bonferroni corrected threshold for gene-wide significance was 3×10^−6^. The overlap of SNPs included in the gene boundaries in our results indicates this is likely to be an overly conservative correction factor [22].

A list of 61 candidate genes with known biological function in the regulation of cortisol was compiled by a panel of experts in the field. All SNPs within and ±50 kb of these genes were examined in the GWAMA results, and gene-based p values were inspected in VEGAS results.

### Exome chip and 1000 Genomes imputed data analysis

Genotypes for the *SERPINA6/SERPINA1* gene region (chr14:94,770,585–94,857,029, build 37) in the CROATIA-Korcula samples (n = 898) were extracted from an Illumina Exome Chip v1 analysis. Genotypes were called in GenomeStudio (Illumina) using the CHARGE Consortium joint calling cluster file (http://www.chargeconsortium.com/main/exomechip) [48]. 1000 Genomes imputation was performed using genotypes from Illumina HumanHap370CNV after quality control (Individual Call Rate 97%, SNP Call Rate 98%, MAF 0.01, HWE 1×10^−6^); prephasing was performed using ShapeIt v2 [49] and imputation using IMPUTE2 [50] and the ALL (Phase 1 integrated release v3, April 2012) reference panel. Associations with plasma cortisol were analysed in GenABEL [51].

### Detailed biochemical studies

More detailed phenotyping was undertaken in samples from 316 participants in the CROATIA-Korcula cohort, comprising 158 age- and sex-matched homozygotes at the top hit variant rs12589136 (53 T/T, 106 G/G; however, one T/T sample had insufficient sample for CBG measurement resulting in 52 T/T and 106 G/G). An additional 160 randomly selected samples had CBG measured, however two samples failed genotyping resulting in an additional 47 T/G and 111 G/G.

Total plasma cortisol was measured with a commercial radioimmunoassay (MP Biomedicals, UK). Total CBG was also measured in CROATIA-Korcula samples using a commercial radioimmunoassay (DiaSource, Louvain-la-Neuve, Belgium). Total cortisol binding capacity was measured using a ligand-saturation assay, with [^3^H]-cortisol (PerkinElmer Life Sciences, Waltham, MA) as the labelled ligand and dextran-coated charcoal to separate the CBG-bound [^3^H]cortisol, as previously described [52]. α1-Antitrypsin was measured with a commercial ELISA (Genway Biotech, San Diego, USA). Unbound free plasma cortisol was calculated from measured total plasma cortisol and total CBG values using Coolens' equation [53]. Free cortisol was also measured by ELISA following equilibrium dialysis. Briefly, dialysis tubing (12–14 kD, Medicell, London, UK) was heated to 80C for 30 min in 2% Na bicarbonate and 1 mM EDTA before overnight dialysis of plasma into phosphate buffered saline containing 1% gelatin at 37C and measurement of dialysed free cortisol by commercial ELISA (Salimetrics Europe Ltd, Newmarket, UK). CBG were also measured, as previously described [23], by ELISAs using monoclonal antibodies 12G2 and 9G12. Antibody 9G12 binds to an epitope in the reactive centre loop (RCL), the elastase cleavage site on CBG, and has been used to infer intact (uncleaved) CBG, whereas antibody 12G2 binds to a distant epitope and measures total (cleaved and uncleaved) CBG.

As CROATIA-Korcula is a population isolate, we used the polygenic and mmscore functions in GenABEL [51]. All regression equations included the first three principal components and kinship matrix derived from GWAS data in this population and used z-scores of the traits. All variables were normalised using log transformation (cortisol, calculated free cortisol, measured free cortisol, CBG, α1-antitrypsin), and reported means are geometric means, the ratio of intact/cleaved was normally distributed.

## Supporting Information

Table S1 Characteristics of participants in cohorts included in genome-wide association meta-analysis and replication for morning plasma cortisol.
(DOCX)

Table S2 Genome-wide data characteristics for discovery cohorts used in meta-analysis.
(DOCX)

Table S3 All SNPs with p-values <5×10^−5^ in discovery genome wide association meta-analysis for morning plasma cortisol.
(DOCX)

Table S4 Top 10 genes identified in VEGAS as associated with morning plasma cortisol from genome wide association meta-analysis with adjustment for age and sex.
(DOCX)

Table S5 VEGAS results for candidate genes based on genome wide association meta-analysis results adjusted for age and sex.
(DOCX)

Table S6 Replication results in individual cohorts and by meta-analysis for association with morning plasma cortisol of SNPs representing independent signals in the SERPINA6/SERPINA1 region discovered in genome-wide association meta-analysis.
(DOCX)

Table S7 Associations with plasma cortisol of variants in SERPINA6/A1 locus on chromosome 14 identified by exome chip in n = 808 subjects from CROATIA-Korcula.
(DOCX)

Text S1 Supporting methods.
(DOCX)
