# Supplementary material for: Genome Wide Association Identifies Common Variants at the SERPINA6/SERPINA1 Locus Influencing Plasma Cortisol and Corticosteroid Binding Globulin
Source: PLoS Genet. 2014 Jul 10;10(7):e1004474. doi: 10.1371/journal.pgen.1004474 (PMC4091794; doi:10.1371/journal.pgen.1004474)
Supplement: Table S1 — Characteristics of participants in cohorts included in genome-wide association meta-analysis and replication for morning plasma cortisol. (DOCX) [file pgen.1004474.s001.docx]

**Table S1. Characteristics of participants in cohorts included in genome-wide association meta-analysis and replication for morning plasma cortisol**

|  |  | Sample size | | Men | Age in years | |  | Plasma cortisol in nmol/l | | Sampling time frame |
| --- | --- | --- | --- | --- | --- | --- | --- | --- | --- | --- |
| Study ID | Full study name | Total | Included | (%) | mean(sd) | range |  | mean(sd) | Range | Hr |
| **Discovery meta-analysis cohorts** | |  |  |  |  |  |  |  |  |  |
| ORCADES | Orkney Complex Disease Study | 889 | 886 | 45.4 | 53.5 (15.7) | 17-97 |  | 765 (315) | 11-3641 | 0830-1030 |
| CROATIA-Korcula | 100001 Dalmations  The Croatian Biobank | 898 | 898 | 36.2 | 56.2 (13.9) | 18-98 |  | 698 (207) | 59-815 | 0800-0900 |
| CROATIA-Split |  | 499 | 496 | 42.9 | 45.0 (14.7) | 18-85 |  | 979 (404) | 94-2831 | 0730-0900 |
| CROATIA-Vis |  | 924 | 892 | 43.5 | 56.4 (15.5) | 18-93 |  | 622 (230) | 64-1820 | 0730-0900 |
| Rotterdam Study | Rotterdam Study | 4797 | 2945 | 43.6 | 71.9 (7.0) | 61-105 |  | 305 (94) | 5-679 | 0800-1100 |
| HBCS1934-44 | Helsinki Birth Cohort 1934-44 Study | 451 | 451 | 36.1 | 60.61 (2.80) | 56 - 67 |  | 393 (120) | 125-990 | 0750-1055 |
| NFBC1966 | Northern Finland Birth Cohort 1966 | 4,936 | 1,192 | 0 | 31(0) | n/a |  | 380 (160) | 40-2370 | 0800-1100 |
| ALSPAC | Avon Longitudinal Study of Parents and Children | 9,912 | 1,567 | 50.3 | 15.43 (0.26) | 14-17 |  | 486 (174) | 58-1683 | 0800-1057 |
| InChianti | Invecchiare in Chianti, aging in Chianti | 1,231 | 1,210 | 44.5 | 68.3 (15.6) | 21-102 |  | 375 (135) | 19-1291 | Before 0900 |
| PIVUS | Prospective Investigation of the Vasculature in Uppsala Seniors | 949 | 919 | 50.2 | 70.2 (0.17) | 69-72 |  | 386 (125) | 31-930 | 0800-1000 |
| PREVEND | Prevention of Renal and Vascular End-stage Disease | 3920 | 1151 | 50.6 | 49.4 (13.0) | 28-75 |  | 442 (201) | 20-1734 | 0800-1100 |
| **Replication cohorts** | |  |  |  |  |  |  |  |  |  |
| ET2DS | Edinburgh Type 2 Diabetes Study | 1066 | 1048 | 51.3 | 67.9 (4.2) | 60-74 |  | 731 (190) | 102-1447 | 0800-0830 |
| Raine | Western Australia Pregnancy Cohort (Raine) | 902 | 797 | 51.9 | 17.1(0.29) | 16.01-18.91 |  | 614 (235) | 36-1654 | Awakening (before 1000) |
| MrOS | Osteoporotic Fractures in Men – Sweden | 929 | 929 | 100 | 75.3 (3.2) | 70-81 |  | 486 (137) | 70-1550 | 0700-1000 |
