# Supplementary material for: Genome Wide Association Identifies Common Variants at the SERPINA6/SERPINA1 Locus Influencing Plasma Cortisol and Corticosteroid Binding Globulin
Source: PLoS Genet. 2014 Jul 10;10(7):e1004474. doi: 10.1371/journal.pgen.1004474 (PMC4091794; doi:10.1371/journal.pgen.1004474)
Supplement: Table S2 — Genome-wide data characteristics for discovery cohorts used in meta-analysis. (DOCX) [file pgen.1004474.s002.docx]

**Table S2. Genome-wide data characteristics for discovery cohorts used in meta-analysis**

|  |  | SNPs  Post-QC |  | Imputation | Lambda | |
| --- | --- | --- | --- | --- | --- | --- |
| Study ID | N |  | Genechip | Program | Pre-QC | Post-QC |
| Orcades | 886 | 2,341,199 | Illumina HumanHap300 | MACH | 1.016 | 0.996 |
| CROATIA-Korcula | 898 | 2,325,159 | Illumina HumanCNV370 | MACH | 1.008 | 1.003 |
| CROATIA-Split | 493 | 2,352,221 | Illumina HumanCNV370 | MACH | 1.001 | 1.001 |
| CROATIA-Vis | 885 | 2,344,271 | Illumina HumanHap300 | MACH | 1.002 | 0.999 |
| Rotterdam Study | 2,945 | 2,377,062 | Version 3  Illumina Infinium II HumanHap 550 | MACH | 0.999 | 1.000 |
| HBCS1934-44 | 451 | 2,359,627 | Modified Illumina 610k | MACH | 1.000 | 1.001 |
| NFBC1966 | 1,190 | 2,279,845 | Illumina HumanCNV370DUO | IMPUTE | 1.484 | 0.999 |
| ALSPAC | 1,567 | 2,377,223 | Illumina HumanHap550K | MACH | 0.993 | 0.995 |
| InChianti | 1,207 | 2,377,020 | Illumina 550K | MACH | 1.014 | 1.013 |
| PIVUS | 919 | 2,479,893 | merged Human Omni Express and MetaboChip | IMPUTE2 | 1.036 | 1.002 |
| PREVEND | 1,151 | 1,971,729 | Illumina Cyto SNP12 v2 array | Beagle 3.3.1 | 1.014 | 1.012 |

Results refer to data used in age and sex adjusted models and post-imputation. Pre-QC lambda results are based on GWAS provided by each cohort for use in meta-analysis. Post QC results were after quality control applied prior to meta-analysis, ie. minor allele frequency (MAF)>2%, call rate >95%, Hardy-Weinberg equilibrium (HWE)> 1x10^-8^ and good imputation quality (MACH R2_HAT>0.30, IMPUTE PROPER_INFO>0.60, BEAGLE INFO>0.30)
