# Supplementary material for: Genome Wide Association Identifies Common Variants at the SERPINA6/SERPINA1 Locus Influencing Plasma Cortisol and Corticosteroid Binding Globulin
Source: PLoS Genet. 2014 Jul 10;10(7):e1004474. doi: 10.1371/journal.pgen.1004474 (PMC4091794; doi:10.1371/journal.pgen.1004474)
Supplement: Table S3 — All SNPs with p-values <5×10^−5^ in discovery genome wide association meta-analysis for morning plasma cortisol. (DOCX) [file pgen.1004474.s003.docx]

**Table S3. All SNPs with p-values <5x10^-5^ in discovery genome wide association meta-analysis for morning plasma cortisol**

|  | **Position (b37)** |  |  |  |  | **Without genomic control** | | |  | **With genomic control** | | |
| --- | --- | --- | --- | --- | --- | --- | --- | --- | --- | --- | --- | --- |
| **Chr** |  | **SNP** | **EAF** | **Effects** | **I^2^** | **Beta** | **se** | **p.value** |  | **Beta** | **se** | **p.value** |
| 14 | 94,793,686 | rs12589136 | 0.22 | +++++++++++ | 0.18 | 0.1 | 0.01 | 3.32E-12 |  | 0.1 | 0.01 | 4.04E-12 |
| 14 | 94,788,341 | rs941599 | 0.22 | +++++++++++ | 0.15 | 0.1 | 0.01 | 3.84E-12 |  | 0.1 | 0.01 | 4.93E-12 |
| 14 | 94,787,288 | rs7161521 | 0.22 | +++++++++++ | 0.15 | 0.1 | 0.01 | 4.03E-12 |  | 0.1 | 0.01 | 5.18E-12 |
| 14 | 94,789,117 | rs2281518 | 0.22 | +++++++++++ | 0.16 | 0.1 | 0.01 | 4.07E-12 |  | 0.1 | 0.01 | 5.22E-12 |
| 14 | 94,827,068 | rs2749527 | 0.49 | ----------- | 0 | -0.08 | 0.01 | 5.21E-11 |  | -0.08 | 0.01 | 6.21E-11 |
| 14 | 94,773,450 | rs11629171 | 0.28 | ++++++++?++ | 0 | 0.09 | 0.01 | 5.53E-10 |  | 0.09 | 0.01 | 6.80E-10 |
| 14 | 94,820,459 | rs2749529 | 0.47 | +++++++++++ | 0.05 | 0.07 | 0.01 | 2.59E-09 |  | 0.07 | 0.01 | 3.01E-09 |
| 14 | 94,816,299 | rs2749530 | 0.54 | ----------- | 0.06 | -0.07 | 0.01 | 3.99E-09 |  | -0.07 | 0.01 | 4.60E-09 |
| 14 | 94,834,575 | rs3762132 | 0.54 | ----------- | 0.2 | -0.07 | 0.01 | 8.48E-09 |  | -0.07 | 0.01 | 9.57E-09 |
| 14 | 94,836,784 | rs1243171 | 0.47 | +++++++++++ | 0.19 | 0.07 | 0.01 | 9.58E-09 |  | 0.07 | 0.01 | 1.08E-08 |
| 14 | 94,829,668 | rs12588394 | 0.23 | +++++++-+++ | 0.08 | 0.09 | 0.01 | 1.24E-08 |  | 0.09 | 0.01 | 1.41E-08 |
| 14 | 94,830,555 | rs3819333 | 0.23 | +++++++-+++ | 0.03 | 0.08 | 0.01 | 1.50E-08 |  | 0.08 | 0.01 | 1.75E-08 |
| 14 | 94,836,529 | rs4900229 | 0.23 | +++++++-+++ | 0.1 | 0.09 | 0.02 | 1.56E-08 |  | 0.09 | 0.02 | 1.80E-08 |
| 14 | 94,835,647 | rs3748319 | 0.23 | +++++++-+++ | 0.1 | 0.09 | 0.02 | 1.57E-08 |  | 0.09 | 0.02 | 1.82E-08 |
| 14 | 94,834,861 | rs3762130 | 0.23 | +++++++-+++ | 0.1 | 0.09 | 0.02 | 1.65E-08 |  | 0.09 | 0.02 | 1.91E-08 |
| 14 | 94,832,016 | rs941595 | 0.78 | -------+--- | 0.06 | -0.08 | 0.01 | 2.66E-08 |  | -0.08 | 0.01 | 3.04E-08 |
| 14 | 94,834,336 | rs1950652 | 0.78 | -------+--- | 0.14 | -0.08 | 0.02 | 3.09E-08 |  | -0.08 | 0.02 | 3.52E-08 |
| 14 | 94,769,476 | rs11621961 | 0.36 | --------?-- | 0 | -0.08 | 0.01 | 3.97E-08 |  | -0.08 | 0.01 | 4.41E-08 |
| 14 | 94,821,237 | rs4905188 | 0.78 | -------+--- | 0.11 | -0.08 | 0.01 | 4.53E-08 |  | -0.08 | 0.01 | 5.17E-08 |
| 14 | 94,816,121 | rs1956174 | 0.78 | -------+--- | 0.13 | -0.08 | 0.01 | 5.09E-08 |  | -0.08 | 0.01 | 5.58E-08 |
| 14 | 94,808,664 | rs909287 | 0.78 | -------+--- | 0.16 | -0.08 | 0.01 | 8.32E-08 |  | -0.08 | 0.01 | 9.43E-08 |
| 14 | 94,808,831 | rs11629326 | 0.23 | +++++++-+++ | 0.12 | 0.08 | 0.01 | 8.33E-08 |  | 0.08 | 0.01 | 9.49E-08 |
| 14 | 94,773,411 | rs2144835 | 0.71 | ++++++++?++ | 0 | 0.08 | 0.01 | 3.38E-07 |  | 0.08 | 0.02 | 3.92E-07 |
| 11 | 103,458,566 | rs1075533 | 0.96 | ++++--+++++ | 0.17 | 0.17 | 0.03 | 7.74E-07 |  | 0.17 | 0.03 | 8.55E-07 |
| 1 | 102,890,127 | rs1340395 | 0.93 | --+-------- | 0.26 | -0.13 | 0.03 | 1.09E-06 |  | -0.13 | 0.03 | 1.23E-06 |
| 1 | 102,890,347 | rs1340396 | 0.93 | --+-------- | 0.26 | -0.13 | 0.03 | 1.14E-06 |  | -0.13 | 0.03 | 1.29E-06 |
| 14 | 94,773,945 | rs8022616 | 0.12 | +-++++++?++ | 0 | 0.1 | 0.02 | 1.72E-06 |  | 0.1 | 0.02 | 1.94E-06 |
| 14 | 74,168,431 | rs6830 | 0.68 | ++++++++++- | 0.53 | 0.06 | 0.01 | 1.94E-06 |  | 0.06 | 0.01 | 2.08E-06 |
| 1 | 102,890,643 | rs1340397 | 0.08 | ++-+++++?++ | 0.34 | 0.13 | 0.03 | 2.15E-06 |  | 0.13 | 0.03 | 2.39E-06 |
| 14 | 74,120,838 | rs11625686 | 0.67 | ++++++++++- | 0.46 | 0.06 | 0.01 | 2.32E-06 |  | 0.06 | 0.01 | 2.56E-06 |
| 14 | 94,815,785 | rs2736883 | 0.41 | -------+--- | 0.15 | -0.06 | 0.01 | 2.93E-06 |  | -0.06 | 0.01 | 3.22E-06 |
| 14 | 94,811,470 | rs941598 | 0.41 | -------+--- | 0.13 | -0.06 | 0.01 | 3.26E-06 |  | -0.06 | 0.01 | 3.59E-06 |
| 1 | 102,885,219 | rs6687748 | 0.08 | ++-+++++?++ | 0.3 | 0.12 | 0.03 | 3.35E-06 |  | 0.12 | 0.03 | 3.70E-06 |
| 14 | 74,113,613 | rs7153752 | 0.67 | ++++++++++- | 0.46 | 0.06 | 0.01 | 3.46E-06 |  | 0.06 | 0.01 | 3.79E-06 |
| 14 | 74,104,127 | rs12433186 | 0.67 | ++++++++++- | 0.47 | 0.06 | 0.01 | 3.70E-06 |  | 0.06 | 0.01 | 4.00E-06 |
| 14 | 74,187,423 | rs7153989 | 0.31 | ------+---+ | 0.5 | -0.06 | 0.01 | 3.72E-06 |  | -0.06 | 0.01 | 4.13E-06 |
| 14 | 94,770,990 | rs2273399 | 0.89 | -+------?-- | 0 | -0.1 | 0.02 | 3.78E-06 |  | -0.1 | 0.02 | 4.18E-06 |
| 14 | 74,134,717 | rs4635279 | 0.33 | ----------+ | 0.48 | -0.06 | 0.01 | 3.87E-06 |  | -0.06 | 0.01 | 4.24E-06 |
| 14 | 74,216,342 | rs887507 | 0.67 | ++++-+++++- | 0.48 | 0.06 | 0.01 | 3.85E-06 |  | 0.06 | 0.01 | 4.28E-06 |
| 14 | 74,105,519 | rs17782076 | 0.67 | ++++++++++- | 0.46 | 0.06 | 0.01 | 3.93E-06 |  | 0.06 | 0.01 | 4.30E-06 |
| 14 | 74,105,925 | rs11628926 | 0.33 | ----------+ | 0.48 | -0.06 | 0.01 | 3.92E-06 |  | -0.06 | 0.01 | 4.30E-06 |
| 14 | 74,104,247 | rs12433224 | 0.67 | ++++++++++- | 0.46 | 0.06 | 0.01 | 3.95E-06 |  | 0.06 | 0.01 | 4.33E-06 |
| 14 | 74,109,990 | rs7156901 | 0.67 | ++++++++++- | 0.46 | 0.06 | 0.01 | 3.95E-06 |  | 0.06 | 0.01 | 4.33E-06 |
| 14 | 74,102,471 | rs11625142 | 0.67 | ++++++++++- | 0.47 | 0.06 | 0.01 | 4.04E-06 |  | 0.06 | 0.01 | 4.42E-06 |
| 14 | 74,157,070 | rs12434616 | 0.33 | ----------+ | 0.48 | -0.06 | 0.01 | 4.08E-06 |  | -0.06 | 0.01 | 4.46E-06 |
| 14 | 74,105,110 | rs8019281 | 0.33 | ----------+ | 0.48 | -0.06 | 0.01 | 4.10E-06 |  | -0.06 | 0.01 | 4.49E-06 |
| 14 | 74,155,356 | rs7150772 | 0.67 | ++++++++++- | 0.46 | 0.06 | 0.01 | 4.16E-06 |  | 0.06 | 0.01 | 4.54E-06 |
| 14 | 74,102,378 | rs11625042 | 0.33 | ----------+ | 0.48 | -0.06 | 0.01 | 4.18E-06 |  | -0.06 | 0.01 | 4.57E-06 |
| 14 | 74,103,616 | rs17182446 | 0.33 | ----------+ | 0.48 | -0.06 | 0.01 | 4.23E-06 |  | -0.06 | 0.01 | 4.62E-06 |
| 1 | 102,876,232 | rs1856774 | 0.08 | ++-+++++?++ | 0.3 | 0.12 | 0.03 | 4.28E-06 |  | 0.12 | 0.03 | 4.70E-06 |
| 14 | 74,162,079 | rs11624921 | 0.33 | ----------+ | 0.48 | -0.06 | 0.01 | 4.39E-06 |  | -0.06 | 0.01 | 4.79E-06 |
| 14 | 74,161,003 | rs12433462 | 0.67 | ++++++++++- | 0.47 | 0.06 | 0.01 | 4.45E-06 |  | 0.06 | 0.01 | 4.85E-06 |
| 14 | 74,178,800 | rs7182 | 0.33 | ----------+ | 0.55 | -0.06 | 0.01 | 4.57E-06 |  | -0.06 | 0.01 | 4.96E-06 |
| 14 | 94,840,201 | rs17090691 | 0.14 | +++++++++++ | 0 | 0.08 | 0.02 | 4.51E-06 |  | 0.08 | 0.02 | 5.02E-06 |
| 14 | 74,162,203 | rs11624954 | 0.67 | ++++++++++- | 0.47 | 0.06 | 0.01 | 4.73E-06 |  | 0.06 | 0.01 | 5.15E-06 |
| 1 | 102,871,320 | rs4908244 | 0.92 | --+-------- | 0.19 | -0.11 | 0.02 | 4.96E-06 |  | -0.11 | 0.02 | 5.45E-06 |
| 1 | 102,869,587 | rs7552083 | 0.92 | --+-------- | 0.19 | -0.11 | 0.02 | 5.02E-06 |  | -0.11 | 0.02 | 5.50E-06 |
| 1 | 102,869,841 | rs10747427 | 0.92 | --+-------- | 0.19 | -0.11 | 0.02 | 5.06E-06 |  | -0.11 | 0.02 | 5.55E-06 |
| 1 | 102,872,668 | rs7519106 | 0.08 | ++-+++++?++ | 0.29 | 0.12 | 0.03 | 5.30E-06 |  | 0.12 | 0.03 | 5.80E-06 |
| 14 | 74,164,718 | rs17182488 | 0.33 | ----------+ | 0.5 | -0.06 | 0.01 | 6.21E-06 |  | -0.06 | 0.01 | 6.72E-06 |
| 20 | 58,134,141 | rs6070847 | 0.16 | ++-++-+++++ | 0.47 | 0.09 | 0.02 | 6.26E-06 |  | 0.09 | 0.02 | 6.78E-06 |
| 1 | 102,871,545 | rs4908247 | 0.08 | ++-+++++?++ | 0.28 | 0.12 | 0.03 | 6.39E-06 |  | 0.12 | 0.03 | 6.96E-06 |
| 14 | 94,841,500 | rs1884549 | 0.14 | +++++++++++ | 0 | 0.08 | 0.02 | 6.40E-06 |  | 0.08 | 0.02 | 7.09E-06 |
| 1 | 102,871,491 | rs4908245 | 0.08 | ++-+++++?++ | 0.28 | 0.11 | 0.03 | 6.64E-06 |  | 0.11 | 0.03 | 7.22E-06 |
| 1 | 102,871,521 | rs4908246 | 0.08 | ++-+++++?++ | 0.28 | 0.11 | 0.03 | 6.85E-06 |  | 0.11 | 0.03 | 7.46E-06 |
| 14 | 74,165,940 | rs12147465 | 0.67 | ++++++++++- | 0.5 | 0.06 | 0.01 | 7.09E-06 |  | 0.06 | 0.01 | 7.65E-06 |
| 10 | 106,826,482 | rs7090949 | 0.74 | -----+---+- | 0.22 | -0.06 | 0.01 | 7.17E-06 |  | -0.06 | 0.01 | 7.77E-06 |
| 1 | 102,866,726 | rs10747426 | 0.92 | --+-------- | 0.17 | -0.11 | 0.02 | 7.19E-06 |  | -0.11 | 0.02 | 7.82E-06 |
| 1 | 102,866,975 | rs1417227 | 0.92 | --+-------- | 0.19 | -0.11 | 0.02 | 7.60E-06 |  | -0.11 | 0.02 | 8.26E-06 |
| 1 | 102,866,321 | rs10874607 | 0.92 | --+-------- | 0.2 | -0.11 | 0.02 | 7.83E-06 |  | -0.11 | 0.02 | 8.50E-06 |
| 1 | 102,865,841 | rs11164499 | 0.92 | --+-------- | 0.2 | -0.11 | 0.02 | 7.85E-06 |  | -0.11 | 0.02 | 8.52E-06 |
| 1 | 102,865,199 | rs10732729 | 0.92 | --+-------- | 0.2 | -0.11 | 0.02 | 7.94E-06 |  | -0.11 | 0.02 | 8.62E-06 |
| 1 | 102,864,742 | rs7541735 | 0.92 | --+-------- | 0.2 | -0.11 | 0.02 | 7.98E-06 |  | -0.11 | 0.02 | 8.67E-06 |
| 1 | 102,864,980 | rs7541959 | 0.92 | --+-------- | 0.2 | -0.11 | 0.02 | 7.98E-06 |  | -0.11 | 0.02 | 8.67E-06 |
| 1 | 102,860,307 | rs10735767 | 0.92 | --+-------- | 0.2 | -0.11 | 0.02 | 8.01E-06 |  | -0.11 | 0.02 | 8.68E-06 |
| 1 | 102,862,202 | rs2066171 | 0.92 | --+-------- | 0.2 | -0.11 | 0.02 | 8.06E-06 |  | -0.11 | 0.02 | 8.74E-06 |
| 1 | 102,861,502 | rs10874605 | 0.92 | --+-------- | 0.2 | -0.11 | 0.02 | 8.12E-06 |  | -0.11 | 0.02 | 8.81E-06 |
| 1 | 102,863,110 | rs1538827 | 0.92 | --+-------- | 0.2 | -0.11 | 0.02 | 8.33E-06 |  | -0.11 | 0.02 | 9.03E-06 |
| 1 | 102,862,847 | rs2066174 | 0.92 | --+-------- | 0.2 | -0.11 | 0.02 | 8.38E-06 |  | -0.11 | 0.02 | 9.08E-06 |
| 10 | 106,826,835 | rs790755 | 0.74 | -----+---+- | 0.19 | -0.06 | 0.01 | 8.48E-06 |  | -0.06 | 0.01 | 9.15E-06 |
| 10 | 106,810,584 | rs790730 | 0.74 | -----+---+- | 0.21 | -0.06 | 0.01 | 8.83E-06 |  | -0.06 | 0.01 | 9.59E-06 |
| 1 | 102,869,403 | rs7517063 | 0.08 | ++-+++++?++ | 0.27 | 0.11 | 0.03 | 8.89E-06 |  | 0.11 | 0.03 | 9.61E-06 |
| 1 | 102,842,543 | rs2376024 | 0.9 | --+--+---+- | 0.3 | -0.1 | 0.02 | 9.44E-06 |  | -0.1 | 0.02 | 1.01E-05 |
| 10 | 106,809,678 | rs790729 | 0.74 | -----+---+- | 0.2 | -0.06 | 0.01 | 9.31E-06 |  | -0.06 | 0.01 | 1.01E-05 |
| 10 | 106,822,139 | rs1670006 | 0.74 | -----+---+- | 0.21 | -0.06 | 0.01 | 9.53E-06 |  | -0.06 | 0.01 | 1.04E-05 |
| 11 | 103,455,243 | rs11225881 | 0.96 | ++++--++?++ | 0.18 | 0.16 | 0.04 | 1.05E-05 |  | 0.16 | 0.04 | 1.10E-05 |
| 14 | 74,203,690 | rs8007548 | 0.68 | ++++-+++++- | 0.49 | 0.06 | 0.01 | 1.02E-05 |  | 0.06 | 0.01 | 1.10E-05 |
| 11 | 103,457,750 | rs730786 | 0.96 | ++++--++?++ | 0.18 | 0.16 | 0.04 | 1.07E-05 |  | 0.16 | 0.04 | 1.12E-05 |
| 7 | 138,690,145 | rs2354973 | 0.5 | +-+++-++?++ | 0 | 0.06 | 0.01 | 1.03E-05 |  | 0.06 | 0.01 | 1.13E-05 |
| 1 | 102,867,477 | rs12736627 | 0.08 | ++-+++++?++ | 0.27 | 0.11 | 0.02 | 1.06E-05 |  | 0.11 | 0.02 | 1.14E-05 |
| 11 | 103,457,980 | rs730785 | 0.04 | ----++--?-- | 0.18 | -0.16 | 0.04 | 1.09E-05 |  | -0.16 | 0.04 | 1.14E-05 |
| 14 | 74,206,855 | rs17182544 | 0.33 | ----+-----+ | 0.5 | -0.06 | 0.01 | 1.07E-05 |  | -0.06 | 0.01 | 1.16E-05 |
| 14 | 94,799,522 | rs2749544 | 0.55 | +++++++-++- | 0.33 | 0.06 | 0.01 | 1.10E-05 |  | 0.06 | 0.01 | 1.18E-05 |
| 1 | 102,867,247 | rs2376029 | 0.08 | ++-+++++?++ | 0.27 | 0.11 | 0.02 | 1.12E-05 |  | 0.11 | 0.02 | 1.21E-05 |
| 14 | 94,768,859 | rs7141205 | 0.19 | --------?-- | 0 | -0.08 | 0.02 | 1.16E-05 |  | -0.08 | 0.02 | 1.26E-05 |
| 11 | 103,453,197 | rs12577850 | 0.04 | ----+?--?-- | 0.24 | -0.17 | 0.04 | 1.24E-05 |  | -0.17 | 0.04 | 1.30E-05 |
| 1 | 102,861,664 | rs12405100 | 0.08 | ++-+++++?++ | 0.28 | 0.11 | 0.02 | 1.22E-05 |  | 0.11 | 0.03 | 1.31E-05 |
| 1 | 102,865,643 | rs10735769 | 0.08 | ++-+++++?++ | 0.25 | 0.11 | 0.02 | 1.21E-05 |  | 0.11 | 0.02 | 1.31E-05 |
| 1 | 102,861,895 | rs4908242 | 0.08 | ++-+++++?++ | 0.28 | 0.11 | 0.02 | 1.23E-05 |  | 0.11 | 0.03 | 1.32E-05 |
| 1 | 102,862,514 | rs2066173 | 0.08 | ++-+++++?++ | 0.27 | 0.11 | 0.02 | 1.26E-05 |  | 0.11 | 0.02 | 1.36E-05 |
| 1 | 102,863,637 | rs7552301 | 0.08 | ++-+++++?++ | 0.27 | 0.11 | 0.02 | 1.28E-05 |  | 0.11 | 0.02 | 1.38E-05 |
| 1 | 102,863,750 | rs7515236 | 0.08 | ++-+++++?++ | 0.27 | 0.11 | 0.02 | 1.30E-05 |  | 0.11 | 0.02 | 1.39E-05 |
| 1 | 102,865,418 | rs10735768 | 0.08 | ++-+++++?++ | 0.27 | 0.11 | 0.02 | 1.31E-05 |  | 0.11 | 0.02 | 1.41E-05 |
| 14 | 74,209,996 | rs17782157 | 0.33 | ----+-----+ | 0.5 | -0.06 | 0.01 | 1.36E-05 |  | -0.06 | 0.01 | 1.46E-05 |
| 11 | 103,463,622 | rs11225894 | 0.96 | ++++--++?++ | 0.12 | 0.16 | 0.04 | 1.45E-05 |  | 0.16 | 0.04 | 1.52E-05 |
| 14 | 94,843,565 | rs11832 | 0.49 | -------+--- | 0.08 | -0.06 | 0.01 | 1.47E-05 |  | -0.06 | 0.01 | 1.56E-05 |
| 21 | 15,709,849 | rs2822584 | 0.94 | +++-++++++- | 0.21 | 0.12 | 0.03 | 1.49E-05 |  | 0.12 | 0.03 | 1.57E-05 |
| 20 | 44,667,904 | rs6017731 | 0.05 | --------?-+ | 0 | -0.13 | 0.03 | 1.55E-05 |  | -0.13 | 0.03 | 1.62E-05 |
| 11 | 103,464,367 | rs11225895 | 0.04 | ----++--?-- | 0.14 | -0.16 | 0.04 | 1.57E-05 |  | -0.16 | 0.04 | 1.64E-05 |
| 1 | 102,843,599 | rs1591960 | 0.06 | ++-+++++?-+ | 0.09 | 0.14 | 0.03 | 1.51E-05 |  | 0.14 | 0.03 | 1.65E-05 |
| 15 | 94,017,062 | rs1473492 | 0.97 | ++-++-++?++ | 0.28 | 0.21 | 0.05 | 1.45E-05 |  | 0.21 | 0.05 | 1.65E-05 |
| 14 | 94,841,670 | rs1884548 | 0.86 | --------+-- | 0 | -0.08 | 0.02 | 1.56E-05 |  | -0.08 | 0.02 | 1.69E-05 |
| 20 | 49,300,890 | rs6122963 | 0.07 | ----------- | 0 | -0.11 | 0.02 | 1.68E-05 |  | -0.11 | 0.02 | 1.88E-05 |
| 11 | 103,452,376 | rs10488762 | 0.97 | ++++-?++?++ | 0.27 | 0.16 | 0.04 | 1.87E-05 |  | 0.16 | 0.04 | 1.97E-05 |
| 14 | 74,192,247 | rs11627457 | 0.28 | ------+---+ | 0.47 | -0.07 | 0.02 | 1.78E-05 |  | -0.07 | 0.02 | 1.97E-05 |
| 14 | 94,842,842 | rs877083 | 0.86 | --------+-- | 0 | -0.08 | 0.02 | 1.81E-05 |  | -0.08 | 0.02 | 1.97E-05 |
| 14 | 94,842,926 | rs877082 | 0.86 | --------+-- | 0 | -0.08 | 0.02 | 1.82E-05 |  | -0.08 | 0.02 | 1.98E-05 |
| 14 | 94,843,083 | rs877081 | 0.86 | --------+-- | 0 | -0.08 | 0.02 | 1.89E-05 |  | -0.08 | 0.02 | 2.05E-05 |
| 14 | 74,100,151 | rs17782064 | 0.34 | ----------+ | 0.48 | -0.06 | 0.01 | 1.94E-05 |  | -0.06 | 0.01 | 2.09E-05 |
| 21 | 15,709,414 | rs7283184 | 0.94 | +++-++++++- | 0.19 | 0.11 | 0.03 | 2.00E-05 |  | 0.11 | 0.03 | 2.11E-05 |
| 16 | 84,419,078 | rs9630649 | 0.78 | +-+++++++++ | 0 | 0.07 | 0.02 | 1.89E-05 |  | 0.07 | 0.02 | 2.12E-05 |
| 10 | 106,792,734 | rs1881224 | 0.74 | -----+---+- | 0.05 | -0.06 | 0.01 | 2.04E-05 |  | -0.06 | 0.01 | 2.18E-05 |
| 17 | 71,258,846 | rs7217509 | 0.21 | ++-++++???+ | 0.36 | 0.1 | 0.02 | 2.03E-05 |  | 0.1 | 0.02 | 2.18E-05 |
| 6 | 133,861,857 | rs7771144 | 0.15 | -++-------- | 0.06 | -0.08 | 0.02 | 2.14E-05 |  | -0.08 | 0.02 | 2.29E-05 |
| 11 | 128,741,739 | rs7116606 | 0.29 | ----------- | 0 | -0.06 | 0.01 | 2.08E-05 |  | -0.06 | 0.01 | 2.33E-05 |
| 6 | 133,861,782 | rs7771096 | 0.15 | -++-------- | 0.06 | -0.08 | 0.02 | 2.23E-05 |  | -0.08 | 0.02 | 2.38E-05 |
| 5 | 9,173,880 | rs40718 | 0.49 | +-+++++++++ | 0 | 0.05 | 0.01 | 2.18E-05 |  | 0.05 | 0.01 | 2.42E-05 |
| 20 | 49,314,690 | rs6126077 | 0.07 | ----------- | 0 | -0.1 | 0.02 | 2.35E-05 |  | -0.1 | 0.02 | 2.61E-05 |
| 6 | 133,861,522 | rs7767007 | 0.14 | -++--+----- | 0.07 | -0.08 | 0.02 | 2.45E-05 |  | -0.08 | 0.02 | 2.62E-05 |
| 20 | 49,318,354 | rs6122965 | 0.07 | ----------- | 0 | -0.1 | 0.02 | 2.43E-05 |  | -0.1 | 0.02 | 2.69E-05 |
| 20 | 49,301,698 | rs17790636 | 0.93 | +++++++++++ | 0 | 0.1 | 0.02 | 2.61E-05 |  | 0.1 | 0.02 | 2.89E-05 |
| 20 | 61,577,515 | rs2427453 | 0.49 | -----+-?--- | 0 | -0.06 | 0.01 | 2.78E-05 |  | -0.06 | 0.01 | 2.92E-05 |
| 2 | 17,455,029 | rs10495661 | 0.76 | +++-+++++++ | 0.03 | 0.06 | 0.01 | 2.74E-05 |  | 0.06 | 0.01 | 2.95E-05 |
| 14 | 94,801,368 | rs996050 | 0.45 | -------+--+ | 0.35 | -0.05 | 0.01 | 2.98E-05 |  | -0.05 | 0.01 | 3.13E-05 |
| 5 | 9,171,202 | rs40719 | 0.49 | +-+++++++++ | 0 | 0.05 | 0.01 | 2.86E-05 |  | 0.05 | 0.01 | 3.20E-05 |
| 6 | 133,858,495 | rs11964505 | 0.14 | -++--+----- | 0.06 | -0.08 | 0.02 | 3.12E-05 |  | -0.08 | 0.02 | 3.32E-05 |
| 10 | 106,789,694 | rs7083262 | 0.74 | -----+---+- | 0.19 | -0.06 | 0.01 | 3.15E-05 |  | -0.06 | 0.01 | 3.37E-05 |
| 7 | 16,691,511 | rs2061471 | 0.33 | +-++++++++- | 0.35 | 0.06 | 0.01 | 3.29E-05 |  | 0.06 | 0.01 | 3.52E-05 |
| 16 | 73,504,491 | rs7192943 | 0.22 | +++++-++?++ | 0.06 | 0.07 | 0.02 | 3.60E-05 |  | 0.08 | 0.02 | 3.84E-05 |
| 20 | 44,642,406 | rs2250889 | 0.04 | --------+-+ | 0 | -0.13 | 0.03 | 3.74E-05 |  | -0.13 | 0.03 | 3.86E-05 |
| 2 | 17,452,120 | rs17477835 | 0.24 | ---+------- | 0.02 | -0.06 | 0.01 | 3.75E-05 |  | -0.06 | 0.01 | 4.04E-05 |
| 4 | 149,570,069 | rs10452224 | 0.44 | ----------- | 0 | -0.05 | 0.01 | 3.55E-05 |  | -0.05 | 0.01 | 4.13E-05 |
| 17 | 11,491,306 | rs11078021 | 0.27 | ++--+---?-- | 0.36 | -0.06 | 0.02 | 3.73E-05 |  | -0.06 | 0.02 | 4.17E-05 |
| 3 | 103,958,244 | rs11717324 | 0.73 | --+-------- | 0 | -0.06 | 0.01 | 3.92E-05 |  | -0.06 | 0.01 | 4.27E-05 |
| 14 | 94,808,760 | rs7161231 | 0.11 | +++++++++++ | 0 | 0.08 | 0.02 | 4.06E-05 |  | 0.08 | 0.02 | 4.33E-05 |
| 4 | 182,662,286 | rs11934518 | 0.89 | ++-++++++++ | 0 | 0.08 | 0.02 | 3.98E-05 |  | 0.08 | 0.02 | 4.43E-05 |
| 14 | 94,842,145 | rs875989 | 0.12 | ++++++++?++ | 0 | 0.09 | 0.02 | 4.12E-05 |  | 0.09 | 0.02 | 4.55E-05 |
| 8 | 108,925,907 | rs17311146 | 0.17 | ++-+++++-++ | 0 | 0.07 | 0.02 | 4.38E-05 |  | 0.07 | 0.02 | 4.67E-05 |
| 20 | 49,293,630 | rs6122960 | 0.93 | +++++++++++ | 0 | 0.1 | 0.02 | 4.40E-05 |  | 0.1 | 0.02 | 4.77E-05 |
| 6 | 137,692,864 | rs1591208 | 0.25 | +++-+++++++ | 0.24 | 0.06 | 0.02 | 4.40E-05 |  | 0.06 | 0.02 | 4.79E-05 |
| 8 | 108,924,771 | rs17311083 | 0.17 | ++-+++++-++ | 0 | 0.07 | 0.02 | 4.53E-05 |  | 0.07 | 0.02 | 4.81E-05 |
| 14 | 94,815,400 | rs2736886 | 0.56 | +++++++-++- | 0.19 | 0.05 | 0.01 | 4.57E-05 |  | 0.05 | 0.01 | 4.89E-05 |
| 11 | 128,740,683 | rs881333 | 0.71 | +++++++++++ | 0 | 0.06 | 0.01 | 4.48E-05 |  | 0.06 | 0.01 | 4.95E-05 |
| 3 | 103,955,623 | rs13325125 | 0.74 | ----------- | 0 | -0.06 | 0.01 | 4.73E-05 |  | -0.06 | 0.01 | 5.13E-05 |
| 4 | 149,570,680 | rs2358462 | 0.56 | +++++++++++ | 0 | 0.05 | 0.01 | 4.48E-05 |  | 0.05 | 0.01 | 5.17E-05 |
