# Supplementary material for: Genome Wide Association Identifies Common Variants at the SERPINA6/SERPINA1 Locus Influencing Plasma Cortisol and Corticosteroid Binding Globulin
Source: PLoS Genet. 2014 Jul 10;10(7):e1004474. doi: 10.1371/journal.pgen.1004474 (PMC4091794; doi:10.1371/journal.pgen.1004474)
Supplement: Table S4 — Top 10 genes identified in VEGAS as associated with morning plasma cortisol from genome wide association meta-analysis with adjustment for age and sex. (DOCX) [file pgen.1004474.s004.docx]

**Table S4. Top 10 genes identified in VEGAS as associated with morning plasma cortisol from genome wide association meta-analysis with adjustment for age and sex**

|  |  |  |  | **P values**^a^ | | |  |  |
| --- | --- | --- | --- | --- | --- | --- | --- | --- |
| **Gene** | **Chr** | **Start** | **Stop** | **Gene** | **Top 10%^b^** | **Best SNP** | **nSNPs** | **Best SNP** |
| SERPINA1 | 14 | 93,912,836 | 93,926,782 | <1.0E-12 | <1.0E-12 | 5.0E-12 | 188 | rs12589136 |
| SERPINA6 | 14 | 93,840,337 | 93,859,441 | 1.0E-06 | <1.0E-12 | 5.0E-12 | 158 | rs12589136 |
| PNMA1 | 14 | 73,248,238 | 73,250,881 | 1.1E-05 | 1.3E-05 | 2.1E-05 | 80 | rs6830 |
| DNAL1 | 14 | 73,181,330 | 73,238,402 | 1.3E-05 | 2.5E-05 | 2.1E-05 | 112 | rs6830 |
| C14orf43 | 14 | 73,251,577 | 73,323,649 | 2.6E-05 | 1.9E-05 | 2.1E-05 | 124 | rs6830 |
| SERPINA10 | 14 | 93,819,402 | 93,829,349 | 3.3E-05 | 3.5E-05 | 5.0E-12 | 129 | rs12589136 |
| ACOT6 | 14 | 73,153,300 | 73,156,345 | 1.3E-04 | 1.3E-04 | 3.0E-05 | 53 | rs11625686 |
| ACOT4 | 14 | 73,128,162 | 73,132,223 | 2.9E-04 | 2.5E-04 | 4.7E-05 | 46 | rs12433186 |
| ZC3HAV1L | 7 | 138,360,991 | 138,371,315 | 5.5E-04 | 5.5E-04 | 1.0E-05 | 87 | rs2354973 |
| FABP2 | 4 | 120,457,852 | 120,462,764 | 5.7E-04 | 6.7E-04 | 1.9E-04 | 128 | rs1814814 |
|  | | | | | | | | |

^a^P value for statistical significance adjusted for multiple testing = 0.05 / 18,000 Genes = 3x10^-6^

^b^ Summary p-value was calculated from 10% of SNPs included in gene boundary with lowest p-values.
