# Supplementary material for: Genome Wide Association Identifies Common Variants at the SERPINA6/SERPINA1 Locus Influencing Plasma Cortisol and Corticosteroid Binding Globulin
Source: PLoS Genet. 2014 Jul 10;10(7):e1004474. doi: 10.1371/journal.pgen.1004474 (PMC4091794; doi:10.1371/journal.pgen.1004474)
Supplement: Table S5 — VEGAS results for candidate genes based on genome wide association meta-analysis results adjusted for age and sex. (DOCX) [file pgen.1004474.s005.docx]

**Table S5. VEGAS results for candidate genes based on genome wide association meta-analysis results adjusted for age and sex**

|  |  |  |  | **P values^a^** | | |  |  |
| --- | --- | --- | --- | --- | --- | --- | --- | --- |
| **Gene** | **Chr** | **Start** | **Stop** | **Gene** | **Top 10%** | **Best SNP** | **nSNPs** | **Best SNP** |
| SERPINA6 | 14 | 93,840,337 | 93,859,441 | <1.0E-12 | <1.0E-12 | 5.04E-12 | 158 | rs12589136 |
| CRHR1 | 17 | 41,217,448 | 41,268,973 | 0.004 | 0.004 | 0.001 | 155 | rs17690703 |
| IL1B | 2 | 113,303,807 | 113,310,827 | 0.014 | 0.013 | 0.002 | 96 | rs11676014 |
| CYP3A4 | 7 | 99,192,539 | 99,219,744 | 0.049 | 0.051 | 0.014 | 44 | rs12333983 |
| POU1F1 | 3 | 87,391,472 | 87,408,427 | 0.077 | 0.077 | 0.007 | 144 | rs17024118 |
| HTR1A | 5 | 63,292,033 | 63,293,302 | 0.100 | 0.100 | 0.004 | 43 | rs17484837 |
| FKBP4 | 12 | 2,774,413 | 2,783,385 | 0.102 | 0.106 | 0.002 | 80 | rs4075718 |
| SRD5A1 | 5 | 6,686,499 | 6,722,675 | 0.114 | 0.104 | 0.007 | 186 | rs6876835 |
| MC2R | 18 | 13,872,042 | 13,905,535 | 0.125 | 0.110 | 0.028 | 172 | rs9959473 |
| MRAP | 21 | 32,585,994 | 32,608,965 | 0.138 | 0.146 | 0.023 | 91 | rs2254251 |
| HSD11B1 | 1 | 207,926,172 | 207,974,918 | 0.142 | 0.138 | 0.009 | 144 | rs4844484 |
| AKR1D1 | 7 | 137,411,735 | 137,453,590 | 0.154 | 0.111 | 0.001 | 126 | rs7802161 |
| AVPR1B | 1 | 204,390,905 | 204,398,105 | 0.249 | 0.246 | 0.042 | 10 | rs28419084 |
| SF1 | 11 | 64,288,653 | 64,302,817 | 0.252 | 0.261 | 0.007 | 41 | rs559566 |
| ACE | 17 | 58,908,165 | 58,928,711 | 0.253 | 0.255 | 0.031 | 61 | rs4311 |
| CYP11B1 | 8 | 143,950,774 | 143,958,238 | 0.267 | 0.260 | 0.002 | 118 | rs5297 |
| CYP17A1 | 10 | 104,580,277 | 104,587,280 | 0.291 | 0.251 | 0.022 | 97 | rs11191425 |
| CYP2R1 | 11 | 14,856,131 | 14,870,327 | 0.292 | 0.302 | 0.052 | 66 | rs1496174 |
| POMC | 2 | 25,237,225 | 25,245,063 | 0.293 | 0.303 | 0.045 | 58 | rs7565877 |
| HMGCR | 5 | 74,668,854 | 74,693,681 | 0.307 | 0.316 | 0.012 | 79 | rs16872523 |
| AVPR1A | 12 | 61,826,482 | 61,832,857 | 0.308 | 0.319 | 0.048 | 96 | rs4763062 |
| MED15 | 22 | 19,191,885 | 19,271,919 | 0.347 | 0.340 | 0.015 | 180 | rs9612171 |
| VDR | 12 | 46,521,586 | 46,585,081 | 0.351 | 0.350 | 0.010 | 168 | rs10783222 |
| H6PD | 1 | 9,217,449 | 9,253,981 | 0.355 | 0.362 | 0.009 | 90 | rs17377443 |
| TNF | 6 | 31,651,328 | 31,654,091 | 0.372 | 0.368 | 0.019 | 172 | rs2071591 |
| KCNMA1 | 10 | 78,299,367 | 79,067,583 | 0.375 | 0.389 | 0.003 | 1115 | rs866864 |
| BDNF | 11 | 27,633,017 | 27,699,872 | 0.388 | 0.408 | 0.049 | 107 | rs7930533 |
| GLP1R | 6 | 39,124,534 | 39,163,498 | 0.435 | 0.439 | 0.022 | 210 | rs2300615 |
| NR1H4 | 12 | 99,391,809 | 99,481,774 | 0.443 | 0.437 | 0.031 | 120 | rs35705 |
| KCNH7 | 2 | 162,936,162 | 163,403,486 | 0.451 | 0.497 | 0.001 | 363 | rs16822702 |
| PRKAR1A | 17 | 64,019,704 | 64,040,505 | 0.463 | 0.492 | 0.040 | 93 | rs12150328 |
| TBX19 | 1 | 166,516,901 | 166,550,288 | 0.476 | 0.463 | 0.060 | 139 | rs1008680 |
| CYP11A1 | 15 | 72,417,155 | 72,447,134 | 0.477 | 0.451 | 0.018 | 113 | rs2930292 |
| NUP62 | 19 | 55,101,893 | 55,124,598 | 0.488 | 0.476 | 0.007 | 80 | rs4801810 |
| HTR7 | 10 | 92,490,555 | 92,607,651 | 0.535 | 0.530 | 0.022 | 230 | rs7904560 |
| DHRS9 | 2 | 169,629,544 | 169,660,923 | 0.580 | 0.575 | 0.031 | 87 | rs2287611 |
| TPH2 | 12 | 70,618,892 | 70,712,488 | 0.595 | 0.585 | 0.022 | 222 | rs17110566 |
| CRH | 8 | 67,251,172 | 67,253,252 | 0.598 | 0.584 | 0.046 | 103 | rs2469298 |
| SRD5A2 | 2 | 31,603,159 | 31,659,544 | 0.616 | 0.612 | 0.096 | 78 | rs614173 |
| PTGES3 | 12 | 55,343,391 | 55,368,345 | 0.633 | 0.631 | 0.151 | 50 | rs4759031 |
| FKBP5 | 6 | 35,649,344 | 35,764,692 | 0.634 | 0.663 | 0.008 | 100 | rs2766534 |
| GLP2R | 17 | 9,670,105 | 9,733,747 | 0.642 | 0.666 | 0.007 | 242 | rs3803854 |
| PCSK1 | 5 | 95,751,874 | 95,794,708 | 0.661 | 0.680 | 0.012 | 133 | rs4326166 |
| CRHR2 | 7 | 30,659,387 | 30,688,665 | 0.698 | 0.697 | 0.005 | 119 | rs12701020 |
| LEP | 7 | 127,668,566 | 127,684,918 | 0.700 | 0.742 | 0.017 | 92 | rs17151983 |
| STAR | 8 | 38,119,374 | 38,127,757 | 0.713 | 0.705 | 0.170 | 35 | rs3015656 |
| HSD11B2 | 16 | 66,022,536 | 66,028,955 | 0.774 | 0.794 | 0.263 | 39 | rs12920590 |
| MC4R | 18 | 56,189,543 | 56,190,981 | 0.776 | 0.786 | 0.041 | 81 | rs17773507 |
| AVP | 20 | 3,011,201 | 3,013,370 | 0.794 | 0.761 | 0.019 | 73 | rs11087565 |
| LEPR | 1 | 65,658,905 | 65,875,410 | 0.806 | 0.812 | 0.092 | 345 | rs17127832 |
| NR3C1 | 5 | 142,637,688 | 142,795,270 | 0.811 | 0.808 | 0.026 | 172 | rs13359372 |
| SLC6A4 | 17 | 25,549,031 | 25,586,841 | 0.823 | 0.808 | 0.052 | 82 | rs11657536 |
| ADCY9 | 16 | 3,952,652 | 4,106,187 | 0.839 | 0.850 | 0.040 | 259 | rs9932389 |
| CYP21A2 | 6 | 32,114,060 | 32,117,398 | 0.885 | 0.879 | 0.088 | 51 | rs34878747 |
| PROP1 | 5 | 177,351,841 | 177,355,849 | 0.902 | 0.921 | 0.117 | 53 | rs13361130 |
| HSD3B2 | 1 | 119,759,295 | 119,767,174 | 0.954 | 0.958 | 0.013 | 152 | rs6667203 |
| POR | 7 | 75,382,355 | 75,454,109 | 0.957 | 0.949 | 0.116 | 69 | rs42183 |
| NR3C2 | 4 | 149,219,364 | 149,583,093 | 0.957 | 0.952 | 0.026 | 396 | rs7699349 |
| ADCY7 | 16 | 48,879,323 | 48,909,544 | 0.960 | 0.976 | 0.146 | 86 | rs9940400 |
| KPNB1 | 17 | 43,082,273 | 43,116,003 | 0.982 | 0.977 | 0.318 | 48 | rs17609248 |
| IL6 | 7 | 22,733,342 | 22,738,145 | 0.987 | 0.987 | 0.072 | 171 | rs2905341 |
| ^a^P value for statistical significance adjusted for multiple testing = 0.05 / 62 Genes = 0.0008 | | | | | | | | |
