# Supplementary material for: Genome Wide Association Identifies Common Variants at the SERPINA6/SERPINA1 Locus Influencing Plasma Cortisol and Corticosteroid Binding Globulin
Source: PLoS Genet. 2014 Jul 10;10(7):e1004474. doi: 10.1371/journal.pgen.1004474 (PMC4091794; doi:10.1371/journal.pgen.1004474)
Supplement: Table S6 — Replication results in individual cohorts and by meta-analysis for association with morning plasma cortisol of SNPs representing independent signals in the SERPINA6/SERPINA1 region discovered in genome-wide association meta-analysis. (DOCX) [file pgen.1004474.s006.docx]

**Table S6. Replication results in individual cohorts and by meta-analysis for association with morning plasma cortisol of SNPs representing independent signals in the *SERPINA6/SERPINA1* region discovered in genome-wide association meta-analysis**

|  |  |  | REPLICATION META | | | | |  | MrOS-Sweden (n=929) | | |  | ET2DS (n=1069) | | |  | Raine (n=797) | | |
| --- | --- | --- | --- | --- | --- | --- | --- | --- | --- | --- | --- | --- | --- | --- | --- | --- | --- | --- | --- |
| SNP ID |  |  | EAF | Beta (95%CI) | P | Effects | |  | EAF | Beta (95%CI) | P |  | EAF | Beta (95%CI) | P |  | EAF | Beta (95%CI) | P |
| rs12589136 | G/T |  | 0.79 | -0.12 (-0.18,-0.06) | 1.92E-4 | | --- |  | 0.80 | -0.11 (-0.23,0.00) | 0.056 |  | 0.79 | -0.23 (-0.33,-0.12) | 2.14E-5 |  | 0.77 | -0.02 (-0.12,0.08) | 0.735 |
| rs11621961 | C/T |  | 0.63 | 0.08 (0.03,0.14) | 0.003 | | +++ |  | 0.65 | 0.04 (-0.06,0.14) | 0.427 |  | 0.60 | 0.09 (0.00,0.17) | 0.055 |  | 0.66 | 0.12 (0.02,0.21) | 0.016 |
| rs2749529 | T/A |  | 0.45 | 0.06 (0.01,0.11) | 0.019 | | +++ |  | 0.47 | 0.01 (-0.08,0.11) | 0.790 |  | 0.44 | 0.1 (0.01,0.18) | 0.021 |  | 0.46 | 0.06 (-0.03,0.15) | 0.172 |
