# Supplementary material for: Genome Wide Association Identifies Common Variants at the SERPINA6/SERPINA1 Locus Influencing Plasma Cortisol and Corticosteroid Binding Globulin
Source: PLoS Genet. 2014 Jul 10;10(7):e1004474. doi: 10.1371/journal.pgen.1004474 (PMC4091794; doi:10.1371/journal.pgen.1004474)
Supplement: Table S7 — Associations with plasma cortisol of variants in SERPINA6/A1 locus on chromosome 14 identified by exome chip in n = 808 subjects from CROATIA-Korcula. (DOCX) [file pgen.1004474.s007.docx]

**Table S7 Associations with plasma cortisol of variants in *SERPINA6/A1* locus on chromosome 14 identified by exome chip in n=808 subjects from CROATIA-Korcula**

| SNP ID | Ch 14 Position (b37) | Gene | Effect | MAF | Beta | Beta SE | P value^a^ |
| --- | --- | --- | --- | --- | --- | --- | --- |
| rs2228541 | 94776221 | SERPINA6 | C/A Ala246Ser | 0.47 | -0.029 | 0.051 | 0.574 |
| rs113418909^b^ | 94780642 | SERPINA6 | A/T Leu115His | 0.022 | -0.70 | 0.17 | 4.51E-5 |
| rs1303 | 94844843 | SERPINA1 | A/C Glu400Asp | 0.29 | -0.0011 | 0.056 | 0.984 |
| rs61761869 | 94844866 | SERPINA1 | G/A Pro393Ser | 0.0012 | 0.33 | 0.72 | 0.641 |
| rs28929474 | 94844947 | SERPINA1 | G/A Glu366Lys | 0.018 | 0.18 | 0.19 | 0.345 |
| rs141620200 | 94845944 | SERPINA1 | C/A Ala308Ser | 0.00062 | -1.46 | 1.01 | 0.148 |
| rs6647 | 94847415 | SERPINA1 | A/G Val237Ala | 0.18 | 0.10 | 0.065 | 0.106 |
| rs709932 | 94849201 | SERPINA1 | G/A Arg125His | 0.16 | 0.078 | 0.068 | 0.251 |
| rs28931570 | 94849388 | SERPINA1 | G/A Arg63Cys | 0.019 | -0.63 | 0.19 | 6.99E-4 |

^a^P value for statistical significance adjusted for multiple testing = 0.05 / 34 variants on exome chip = 0.0015

^b^Previously identified as the Leuven mutation [1]. This table presents data for 808 participants in whom quality control was passed for all exome chip variants; Table 2 presents data for 820 participants for whom quality control was passed for rs113418909.
