# Supplementary material for: Genome Wide Association Identifies Common Variants at the SERPINA6/SERPINA1 Locus Influencing Plasma Cortisol and Corticosteroid Binding Globulin
Source: PLoS Genet. 2014 Jul 10;10(7):e1004474. doi: 10.1371/journal.pgen.1004474 (PMC4091794; doi:10.1371/journal.pgen.1004474)
Supplement: Text S1 — Supporting methods. (DOCX) [file pgen.1004474.s008.docx]

**Text S1 Supporting methods**

**Descriptions of individual cohorts contributing to the CORNET consortium**

**ORCADES Study**

The Orkney Complex Disease Study (ORCADES) is a study based in Orkney, Scotland designed to examine genetic determinants of numerous quantitative phenotypic traits. The study population of n=2078, aged 17-100 years was recruited from a genetic isolated population resource between 2005-2011, of which 886 had both gwas data and morning plasma cortisol measures. Plasma samples were collected after overnight fast between 0830 and 1030 h. Plasma cortisol was measured by RIA (MP Biomedicals, Cambridge, UK).

**Croatia-Korcula study**

The CROATIA-Korcula study, Croatia, is a family-based, cross-sectional study in the isolated island of Korcula that included 965 examinees aged 18-95. Blood samples were collected in 2007 along with many clinical and biochemical measures and lifestyle and health questionnaires. Plasma cortisol was measured by RIA (MP Biomedicals, Cambridge, UK).

**Croatia-Split study**

The CROATIA-Split study, Croatia, is a population-based, cross-sectional study in the Dalmatian City of Split that so far includes 1000 examinees aged 18-95. Blood samples were collected in 2009-2011 along with many clinical and biochemical measures and lifestyle and health questionnaires. Plasma cortisol was measured by RIA (MP Biomedicals, Cambridge, UK).

**Croatia-Vis study**

The CROATIA-Vis study, Croatia, is a family-based, cross-sectional study in the isolated island of Vis that included 1,056 examinees aged 18-93. Blood samples were collected in 2003 and 2004 along with many clinical and biochemical measures and lifestyle and health questionnaires. Plasma cortisol was measured by RIA (MP Biomedicals, Cambridge, UK).

**Rotterdam study**

The Rotterdam Study (RS) is an ongoing population-based cohort study of risk factors for chronic diseases in the elderly which includes approximately 8,000 participants who live in Rotterdam. Detailed information on design, objectives and methods has been presented elsewhere [1]. The third study wave (1997-1999) was used in the current study (N=4797). In total, 2945 had serum cortisol levels and genotype data. The Medical Ethics Committee of the Erasmus Medical Centre approved the Rotterdam Study and written informed consent was obtained from all participants. Cortisol was measured by RIA (DPC, Los Angeles, CA, USA).

**Helsinki Birth Cohort Study 1934-1944**

The Helsinki Birth Cohort Study (HBCS) is composed of 8,760 individuals born between the years 1934-44 in one of the two main maternity hospitals in Helsinki, Finland. Between 2001 and 2003, a randomly selected sample of 928 males and 1075 females participated in a clinical follow-up visit with a focus on cardiovascular, metabolic and reproductive health, cognitive function and depressive symptoms. Blood samples were collected in the visit along with various other biochemical and questionnaire-based data. There were 451 women and men (36.1% men) with valid genotype and phenotype data. The mean age of the participants was 60.6 years (SD=2.8). The HBCS research protocol was approved by the Institutional Review Board of the National Public Health Institute and all participants have signed an informed consent. Cortisol was measured on an immunoanalyser (Bayer Inc., Garytown, NY, USA)

**Northern Finland Birth Cohort 1966 (NFBC1966)**

The Northern Finland Birth Cohort 1966 study was initiated in 1965 by enrolling mothers living in the two Northernmost provinces of Finland (Oulu and Lapland) and with expected dates of delivery in 1966 [2]. Altogether 12,231 children were born into the cohort, 12,058 of them live-born. The original data have been supplemented by data collected with postal questionnaires at the ages of 1, 14 and 31 years and various hospital records and national register data. At 31 years of age, those living in the original target area (Northern Finland) or in the capital (Helsinki) area were invited to a clinical examination, in which 71% (N=6033) participated. Blood samples were drawn and DNA was extracted successfully for 5753 subjects. Serum cortisol was analysed for a subset of the study participants (N=1192). All participants gave written informed consent and ethical committees of University of Oulu and the Northern Ostrobothnia Hospital District have approved the study. Cortisol was measured by RIA (Orion Diagnostica, Oulunsalo, Finland).

**Avon Longitudinal Study of Parents and Children**

ALSPAC recruited pregnant women resident in Avon, UK with expected dates of delivery 1st April 1991 to 31st December 1992. In approximately 1998, the initial sample was expanded by recruiting eligible cases who did not join the study originally. Further details are published previously [3]. The total sample size for analyses using any data collected after the age of seven is from 15,247 pregnancies, resulting in 14,701 children alive at 1 year of age. Samples for the studies reported here were obtained from 1,567 for participants who had attended before 1100h. All the data are available through a fully searchable data dictionary at <http://www.bris.ac.uk/alspac/researchers/data-access/data-dictionary>. Ethical approval for the study was obtained from the ALSPAC Ethics and Law Committee and the Local Research Ethics Committees. Plasma cortisol was measured by RIA (MP Biomedicals, Cambridge, UK).

**InCHIANTI study**

The InCHIANTI study is a population-based epidemiological study aimed at evaluating factors that influence mobility in the older population living in the Chianti region of Tuscany, Italy. All participants were >21 years of age and of white European origin. Cortisol was measured by RIA (Active Cortisol RIA, DSL-2100; Diagnostic Systems Laboratories, Webster, TX, USA).

**PIVUS study**

The participants in the Prospective Investigation of the Vasculature in Uppsala Seniors (PIVUS) study were randomly sampled from all men and women at age 70 living in Uppsala County in 2001. Of the 2025 individuals invited, 1016 participated. The participants underwent a medical examination including a detailed questionnaire on lifestyle and socioeconomic factors, fasting blood sampling, blood pressure measurement and anthropometric measurements. Blood and plasma samples have been frozen until analysis, and blood tests performed include a wide variety of traditional and more recent CVD risk factors, along with DNA extraction. Cortisol was measured on an immunoanalyser (Modular E170, Roche Diagnostics, Mannheim, Germany).

**PREVEND study**

The Prevention of Renal and Vascular End-stage Disease (PREVEND) study is an ongoing prospective study investigating the natural course of increased levels of urinary albumin excretion and its relation to renal and cardiovascular disease. Details of the protocol are described at [www.prevend.org](http://www.prevend.org/). Cortisol was measured on an Abbot Axsym analyser using a Fluorescence Polarization Immunoassay (FPIA)

**Edinburgh Type 2 Diabetes study**

The ET2DS recruited a randomly-selected cohort of 1066 men and women aged 60-74 years with type 2 diabetes resident in Lothian, UK. Plasma samples and DNA used in the current analysis were collected on all participants in 2006/7. Further details have been published previously [4]. Plasma cortisol was measured by RIA (MP Biomedicals, Cambridge, UK).

**Raine study**

The Western Australia Pregnancy Cohort (Raine) recruited 2,900 pregnancies from King Edward Memorial Hospital between 1989 and 1991 as part of a randomised controlled trial which evaluated repeated ultrasounds in pregnancy. Detailed data were collected throughout pregnancy and at follow-ups carried out at ages 1, 2, 3, 6, 8, 10, 14 and 17 years. Between 2006 and 2009, specimens were collected from 1,408 study participants in the home environment as part of the 17-year follow-up. Study participants received a home visit from a research nurse on weekdays and fasted blood was drawn shortly after awakening; all samples were collected before 1000h. Cortisol was measured by RIA (GamaCoat cortisol RIA, DiaSorin, MN, USA )

**MrOS-Sweden study**

The Osteoporotic Fractures in Men (MrOS) study is a multicenter, prospective study including 3,014 elderly men in Sweden, Hong Kong (~2,000), and the United States (~6,000). The MrOS Sweden cohort consist of three sub-cohorts from three different Swedish cities (n=1,005 in Malmo, n=1,010 in Göteborg, and n=999 in Uppsala). In this study, only participants from Göteborg were used. Study subjects were randomly identified using national population registers, contacted and asked to participate. To be eligible for the study, the subjects had to be able to walk without assistance, provide self-reported data, and sign an informed consent; there were no other exclusion criteria. The study was approved by the ethics committee at the University of Gothenburg. Informed consent was obtained from all study participants. Cortisol was measured by immunoanalyser (Elecsys, Roche Diagnostics Scandinavia AB).

**SUPPLEMENTARY REFERENCES**

1. Hofman A, van Duijn CM, Franco OH, Ikram MA, Janssen HL et al. (2011) The Rotterdam Study: 2012 objectives and design update. Eur J Epidemiol 26: 657-686.

2. Rantakallio P (1969) Groups at risk in low birth weight infants and perinatal mortality. Acta Paediatr Scand 193: Suppl.

3. Boyd A, Golding J, Macleod J, Lawlor DA, Fraser A et al. (2013) Cohort Profile: The 'Children of the 90s'--the index offspring of the Avon Longitudinal Study of Parents and Children. Int J Epidemiol 42: 111-127.

4. Price JF, Reynolds RM, Mitchell RJ, Williamson RM, Fowkes FG et al. (2008) The Edinburgh Type 2 Diabetes Study: study protocol. BMC Endocr Disord 8: 18.
